# Clinical significance, tumor immune landscape and immunotherapy responses of ADAR in pan-cancer and its association with proliferation and metastasis of bladder cancer

**DOI:** 10.18632/aging.204853

**Published:** 2023-07-06

**Authors:** Hao Yu, Kexin Bai, Yidong Cheng, Jiancheng Lv, Qiang Song, Haiwei Yang, Qiang Lu, Xiao Yang

**Affiliations:** 1 Department of Urology, The First Affiliated Hospital of Nanjing Medical University, Nanjing 210029, PR China; 2 Institute of Urology and Andrology, Nanjing Medical University, Nanjing 210029, PR China

**Keywords:** ADAR, cancer, immunotherapy, bladder cancer, tumor immune microenvironment

## Abstract

Background: ADAR is an enzyme involved in adenosine-inosine RNA editing. However, the role of ADAR in tumorigenesis, progression, and immunotherapy has not been fully elucidated.

Methods: The TCGA, GTEx and GEO databases were extensively utilized to explore the expression level of ADAR across cancers. Combined with the clinical information of patients, the risk profile of ADAR in various cancers was delineated. We identified pathways enriched in ADAR and their related genes and explored the association between ADAR expression and the cancer immune microenvironment score and response to immunotherapy. Finally, we specifically explored the potential value of ADAR in the treatment of the bladder cancer immune response and verified the critical role of ADAR in the development and progression of bladder cancer through experiments.

Results: ADAR is highly expressed in most cancers at both the RNA and protein level. ADAR is associated with the aggressiveness of some cancers, especially bladder cancer. In addition, ADAR is associated with immune-related genes, especially immune checkpoint genes, in the tumor immune microenvironment. Moreover, ADAR expression is positively correlated with tumor mutation burden and microsatellite instability in a variety of cancers, indicating that ADAR could be used as a biomarker of immunotherapy. Finally, we demonstrated that ADAR is a key pathogenic factor in bladder cancer. ADAR promoted proliferation and metastasis of bladder cancer cells.

Conclusion: ADAR regulates the tumor immune microenvironment and can be used as a biomarker of the tumor immunotherapy response, providing a novel strategy for the treatment of tumors, especially bladder cancer.

## INTRODUCTION

After decades of unremitting efforts by medical scientists, great progress has been made in cancer treatment strategies and clinical outcomes. However, many patients remain stranded in the shadow of drug resistance, disease progression, relapse, and eventual death [1]. Among a variety of treatment modalities, antitumor immunotherapy has received sufficient attention in recent years and has achieved considerable survival benefits in the treatment of multiple cancers, such as breast cancer, melanoma, lung cancer and colorectal cancer [2–5]. Common antitumor immunotherapy methods include therapeutic monoclonal antibody immunotherapy, immune checkpoint inhibitor (ICI) therapy, adoptive cell therapy, oncolytic virus therapy, and tumor vaccines [6, 7]. Among them, the development of immune checkpoint inhibitors represented by PD-1 inhibitors has attracted particular attention. Since only a minority of patients respond to ICIs, the identification of predictive markers and mechanisms of immunotherapy resistance are the subject of intensive research [6, 8]. Therefore, it is necessary to continuously improve the understanding of the pathogenesis of cancer and constantly improve the exploration of the characteristics of tumor genomics and the immune microenvironment to predict and improve the specificity and sensitivity of ICIs to tumor patients and reduce their toxic effects.

As a highly conserved group of enzymes, the adenosine deaminase acting on RNA (ADAR) family mediates adenosine to inosine (A-to-I) RNA editing, by which adenosines are selectively converted to inosines in double-stranded RNA (dsRNA) substrates [9]. However, the overall biological effects of ADAR remain largely unknown. There are three types of ADAR, ADAR1-3, in mammalian cells. Among them, ADAR1 was the first identified ADAR protein and has been extensively studied [10, 11]. Studies have shown that the abnormal expression and dysfunction of ADAR1 may have oncogenic or tumor suppressive effects, affecting tumor proliferation, invasion and response to immunotherapy [12]. For example, early-stage lung cancer patients with ADAR1 amplification often suffer a poor prognosis [13]. In addition, the abnormal upregulation of ADAR1 can promote the proliferation of triple-negative breast cancer cells [14]. Although the functions of ADAR in human cancers have been intensively studied, the specific roles and molecular mechanisms of ADAR in the tumor microenvironment and antitumor immunotherapy remain largely enigmatic.

Given the significance of ADAR, we performed comprehensive bioinformatics analysis and *in vitro* experiments to explore the important role and special molecular mechanisms of ADAR across cancers. First, by integrating information from multiple databases, we found that ADAR is abnormally upregulated in most cancers, including at the transcriptional and protein levels, and is associated with the malignancy and prognosis of a variety of cancers. Subsequently, we explored ADAR mutations, associated genes, and potential molecular pathways across cancers. Notably, we identified the relationship between ADAR and the tumor microenvironment (TME), ICIs, and their regulatory role in immune infiltration and immunotherapy. Finally, we independently investigated the role of ADAR in BLCA and its potential as an immunotherapeutic target, which was validated by a series of experiments. Our findings suggest that ADAR can be used as a biomarker to predict the prognosis of patients with BLCA.

## MATERIALS AND METHODS

### Data collection and processing

Pan-cancer sequencing data and clinicopathological information were downloaded from the TCGA (https://portal.gdc.cancer.gov/), GEO (https://www.ncbi.nlm.nih.gov/geo/), and GTEx databases (https://www.gtexportal.org/). To minimize batch effects, we used transcripts per million (TPM) and normalized them by log2 transformation on the same sequencing platform. The ADAR protein profiles were obtained from the UALCAN database (http://ualcan.path.uab.edu/). ADAR gene mutation data were obtained from the cBioPortal database (http://www.cbioportal.org/). Differences in ADAR mRNA expression between normal and tumor samples were analyzed using the “limma” R package among TCGA, GEO, and GTEx data. ADAR single-cell analysis was analyzed by using HPA. For two groups of *t*-tests, *p* < 0.05 indicated that the expression difference between tumor and normal tissues was statistically significant.

### Relationship with histological grades, cox regression analysis, and survival analysis

Based on the mRNA sequencing data of ADAR in the TCGA database and the clinical information of patients, an independent sample *t*-test was used to analyze the expression difference of ADAR between different histological grades in pan-cancer. Cox regression analysis was used to investigate the relationship between ADAR expression and overall survival (OS), disease-specific survival (DSS), progression-free survival (PFS), and disease-free survival (DFS) in each cancer type. The “forest plot”, “ggrisk”, “survminer”, “survival” and “timeROC” R packages were utilized to visualize the survival analysis. The log-rank test was used to compare differences in survival between these groups. TimeROC (v 0.4) analysis was used to compare the predictive accuracy of ADAR mRNA. For Kaplan-Meier curves, *p*-values and hazard ratios (HRs) with 95% confidence intervals (CIs) were generated by log-rank tests and univariate Cox proportional hazards regression. *P* < 0.05 was considered statistically significant.

### Analysis of ADAR genomic alterations

The genomic mutational landscape of ADAR across cancers was obtained from the cBioPortal database, including alteration frequency, copy number alterations, and mutations. The mutation sites of ADAR are displayed through the schematic diagram or 3D (three-dimensional) structure of the “mutation” module.

### Protein–protein interaction (PPI) network construction and functional enrichment analysis

Through the String database (https://string-db.org/), the top 50 genes that have been verified by experiments to interact with ADAR were obtained and displayed. Subsequently, we obtained the top 100 genes significantly associated with ADAR across cancers via the GEPIA 2 website (http://gepia2.cancer-pku.cn/#index). The key gene sets obtained from the two databases were intersected to obtain the optimal gene(s). In addition, we systematically analyzed the 5 genes with the highest correlation scores. Finally, Gene Ontology (GO), Kyoto Encyclopedia of Genes and Genomes (KEGG) and gene set enrichment analyses (GSEA) were performed to identify the molecular functions and pathways enriched in these genes. *P* < 0.05 was considered statistically significant.

### Analysis of immune infiltration and immune-related genes

The ESTIMATE algorithm was used to explore the infiltration level of immune cells and stromal cells, and the stromal score and immune score were calculated. In addition, Spearman correlation analysis was used to generate correlation heatmaps showing the association between ADAR and immunomodulators (immune-stimulators, MHC genes, chemokines, and chemokine receptors) and immune checkpoint genes. The correlation between ADAR gene expression and tumor mutation burden/microsatellite instability (TMB/MSI) was analyzed by the Spearman method using the “ggstatsplot” R package.

### Bioinformatics exploration of ADAR in BLCA

The TCGA-BLCA samples were divided into a high expression group and a low expression group according to the median value of ADAR expression. The “limma” package in R software was used to study the differentially expressed mRNAs. Adjusted *P* < 0.05 and |Log2 (Fold Change)| >1 were defined as the threshold for the differential expression of mRNAs. The mutation data were downloaded and visualized using the “maftools” package in R software. Oncoplot showed the differences between the somatic landscapes of the two cohorts of BLCA. To further confirm the underlying function of potential targets, the data were analyzed by functional enrichment (GO and KEGG).

We used the Timer 2.0 (http://timer.cistrome.org/) database to characterize the immune cell infiltration landscape in the 6 TCGA-BLCA samples with the highest (TCGA-FD-A3SR-01, TCGA−FD−A3SR−01, TCGA−GC−A3RB−01) and the lowest ADAR expression (TCGA-CF-A3MI-01, TCGA−CF−A47T−01, and TCGA−ZF−A9R4−01). Immunophenograms were constructed to visualize the immunophenotypes of samples in The Cancer Immunome Atlas (TCIA; https://www.tcia.at/home) database. The immunophenogram enables the calculation of an aggregated score, the immunophenoscore (IPS), based on the expression of major determinants, identified by a random forest approach. These factors were classified into four categories: MHC molecules (MHC), immunomodulators (CP), effector cells (EC), and suppressor cells (SC). The database notes that the IPSs were calculated on a 0-10 scale based on the expression of representative genes or gene sets of the immunophenogram. Sample z scores were positively weighted according to stimulator cell type and negatively weighted according to suppressor cell type and then averaged [15]. Subsequently, the IPS was also calculated for all patients in TCGA-BLCA, grouped according to ADAR expression. A potential immune checkpoint blockade (ICB) response was predicted with the Tumor Immune Dysfunction and Exclusion (TIDE) algorithm [16]. Two immunotherapy cohorts, IMvigor210 (*n* = 348) and GSE176307 (*n* = 90), were used to compare ADAR expression levels between groups with different responses to immune checkpoint blockades.

### Clinical specimens

Bladder cancer tissues and their matched para-carcinoma tissues were taken from BLCA patients who underwent surgery at the First Affiliated Hospital of Nanjing Medical University from 2016 to 2019. The deadline for follow-up was June 2022. All patients signed the informed consent form before the use of clinical materials. The Ethics Committee of the First Affiliated Hospital of Nanjing Medical University approved the protocol used in this study.

### RNA isolation and qRT PCR

Total RNA was extracted from bladder cancer tissues and cell lines using TRIzol reagent (Invitrogen, Thermo-Fisher Scientific, USA) according to the manufacturer’s instructions. HiScript II (Vazyme, China) was used to synthesize cDNA. qRT-PCR of mRNA was performed on a StepOne Plus real-time PCR system (Applied Biosystems, USA). Each sample was repeated three times, and the data were analyzed by comparing CT values. Primer sequences included ADAR: ′F: ATCAGCGGGCTGTTAGAATATG′ and ′R: AAACTCTCGGCCATTGATGAC′ and β-Actin: ′F: GGAGATTACTGCCTGGCTCCA′ and ′R: GACTCATCGTACTCCTGCTTGCTG′, purchased from TSINGKE Biological Technology (Beijing, China). We calculated multiple changes in mRNA expression by the 2^−ΔΔCT^ method.

### Western blot

Total tissue and cellular protein were dissolved in RIPA buffer (Sigma, USA) containing a protease inhibitor. The extracted proteins were quantified by bicinchoninic acid (BCA) analysis (Beyotime, China). The protein extracts were isolated by 10% SDS-PAGE and transferred to polyvinylidene fluoride (PVDF) membranes (Millipore, USA). High-affinity anti-ADAR1 antibodies (1:1000, Abcam, USA) and anti-β-actin antibodies (1:1000, Cell Signaling Technology, USA) were used. After incubation, the membrane was incubated with a secondary antibody (1:5000, Cell Signaling Technology, USA) conjugated with peroxidase (HRP). After cleaning, the signal was detected using a chemiluminescence system (Millipore, USA) and analyzed using Image Lab Software (Bio-Rad, USA).

### Tissue microarray (TMA) and immunohistochemistry (IHC)

TMA was constructed from 180 bladder cancer tissues. Microwave heating was used to isolate the antigens. After dipping in 3% H_2_O_2_ for 10 min, the slides were treated with ADAR (1:200; Abcam, USA) at 4°C overnight. Afterward, HRP-conjugated antibody was used to treat the slides at room temperature for 30 min. Images were captured and recorded under a microscope (Nikon Corporation, Japan). Standard staining protocols were used. Stained tissues were scored for staining intensity (SI) and the percentage of positive cells (PP). SI was scored on a scale of 0–3 (0, negative staining; 1, weak staining; 2, moderate staining; 3, strong staining), and PP was scored according to five categories: 0 (0% positive cells), 1 (<10%), 2 (11–50%), 3 (51–80%) or 4 (>80% positive cells). The total score was calculated by multiplying the SI and PP scores, ranging from 0–12. Two pathologists who were blinded to the clinical parameters provided the respective scores. Different scores were divided into low-staining (0–7) and high-staining (8–12) groups.

### Cell culture

BLCA cell lines (T24, BIU87, J82, 253J, 5637, RT4) and one human ureteral epithelial immortalized cell line (SV-HUC-1) were purchased from the Typical Culture Collection Center of the Chinese Academy of Sciences (Shanghai, China), and 10% fetal bovine serum (FBS; Biological Industries, Israel) and 1% penicillin/streptomycin (Gibco, Thermo Fisher Scientific, USA) were included in the study. All cell lines were cultured at 37°C in a humidified incubator containing 5% CO_2_.

### Transfection

Lentiviruses constructed for ADAR knockdown were obtained from OBiO Technology Corp., Ltd. (China). Cells were plated in 6-well dishes until 30% confluence was reached, infected with ADAR overexpression lentivirus (pcSLenti-EF1-EGFP-P2A-Puro-CMV-ADAR-3xFLAG-WPRE, termed as ADAR), a negative control (pcSLenti-EF1-EGFP-P2A-Puro-CMV-MCS-3xFLAG-WPRE, termed as NC); ADAR knockdown lentivirus (pSLenti-U6-shRNA(ADAR)-CMV-EGFP-F2A-Puro-WPRE, termed as shADAR-1 and shADAR-2), and scramble control (pSLenti-U6-shRNA(NC)-CMV-EGFP-F2A-Puro-WPRE, termed as shNC) in bladder cancer cell T24 and BIU87. Structures of stably transduced cells were generated by selection using puromycin (2.5 μg/ml) for 1 week.

### Cell proliferation and colony formation assay

For the cell proliferation assay, cells were evenly spread in a 96-well plate at a density of 2000 cells/well. At 24, 48, 72 and 96 hours after inoculation, the cells were incubated in 10 μl/well CCK-8 diluted for 1 hour. The absorbance was measured at 450 nm with a microplate reader following incubation at 37°C for 1 h according to the manufacturer’s instructions. For the colony formation assay, T24 and BIU87 cells were inoculated on 6-well plates at a rate of 1000 cells/well and incubated in 5% CO_2_ at 37°C for 2 weeks. After fixation with methanol, the cells were stained with 0.1% crystal violet for 30 minutes, and then the colonies were imaged and counted.

### Transwell cell invasion assay

Transfected cells were seeded into the upper chambers with serum-free medium coated with Matrigel (BD Biosciences, USA) for the invasion assay. Medium containing 20% FBS was added to the bottom chamber. After incubation for 24 h at 37°C, cells attached to the upper surface of the membrane were carefully removed with a cotton swab, and cells attached to the lower surface of the membrane were fixed with 10% formalin, stained with crystal violet for 30 min at room temperature and counted.

### Wound healing assay

To determine the effect of ADAR on cell migration, we uniformly inoculated transfected T24 and BIU87 cells in a 6-well plate. When the cell density reached 90-95%, a 200 μl pipette tip was used to draw a straight wound through the cell layer. The cells were washed with phosphate buffered saline (PBS) to remove the isolated cells and kept at 37°C in a humidified incubator containing 5% CO_2_. A digital camera system (Olympus, Tokyo, Japan) was used to take images of wound closure at 0 and 24 hours.

### Statistical analysis

The Wilcoxon rank-sum test was used to compare the differences between the two groups. The K-W test was performed to compare three or more groups. All statistical analyses were performed using R 4.1.0. Statistical significance was set at a two-sided *p*-value <0.05.

### Data availability statement

The original contributions presented in the study are included in the article/Supplementary Materials. Further inquiries can be directed to the corresponding authors.

## RESULTS

### Upregulated expression of ADAR in cancers

**Figure 1:**
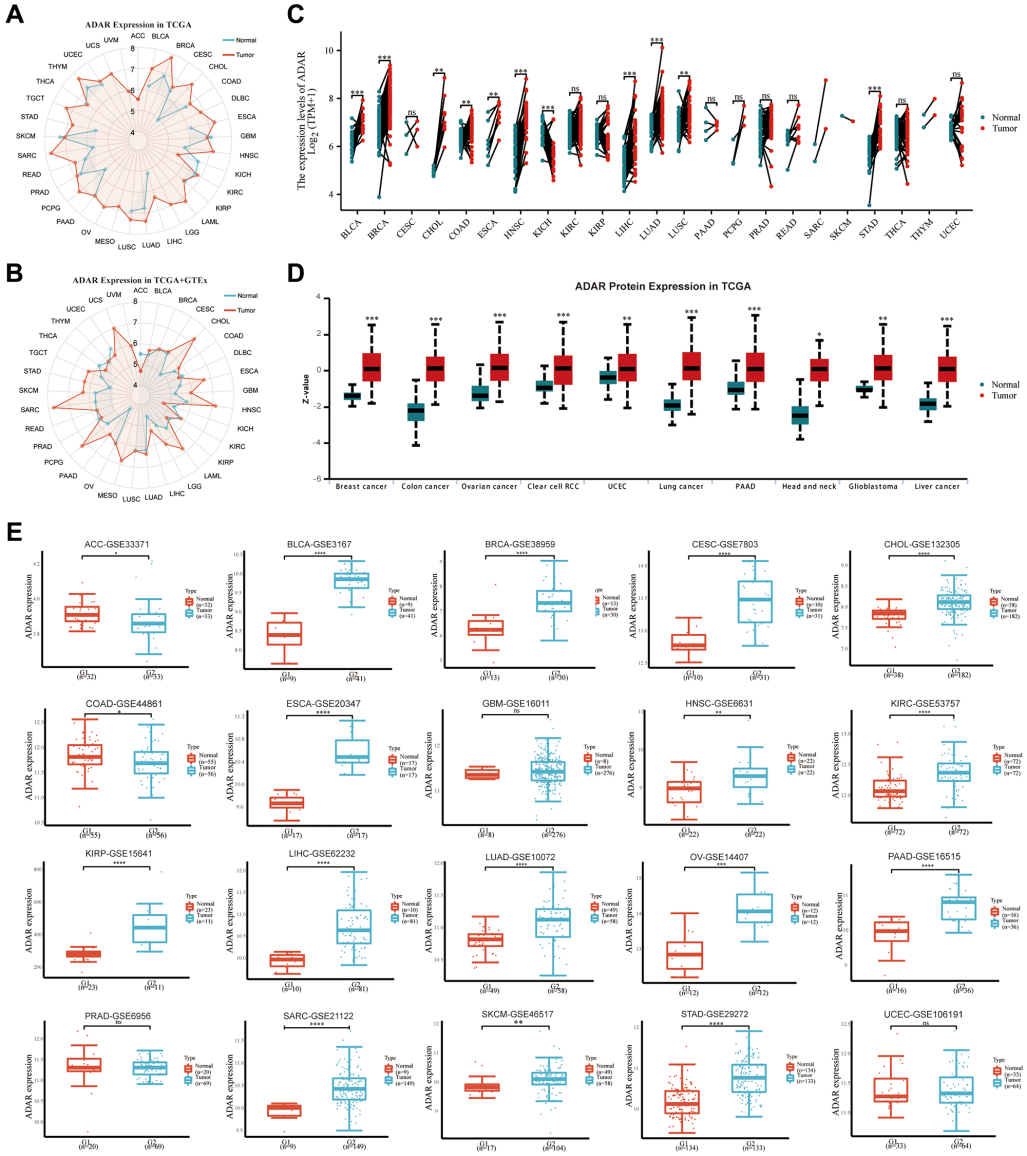
**ADAR is upregulated in most cancers.** (**A**) ADAR expression of pan-cancer in TCGA database. (**B**) ADAR expression of pan-cancer in TCGA database combined with GTEx database. (**C**) Pairing difference analysis of ADAR in TCGA database. (**D**) ADAR protein levels are significantly elevated in a variety of cancers. (**E**) ADAR expression of pan-cancer in GEO database.

We first illustrated the mRNA expression of ADAR in normal versus cancer tissues in pan-cancer using the TCGA database. The results showed that ADAR expression was upregulated in most cancers but downregulated only in GBM, KICH, and SKCM (Figure 1A). Since there were few normal samples included in the TCGA database, we added samples from the GTEx database and drew the expression of ADAR again. The results of our secondary analysis showed that the expression trend of ADAR was basically consistent with the results of the TCGA database, but there were some differences. For example, the expression of ADAR was upregulated in SKCM, THCA, and UCEC (Figure 1B). The results of paired difference analysis in the TCGA cohort showed that ADAR was generally highly expressed across cancers (Figure 1C). In addition, the UALCAN database was used to study the differences in ADAR protein levels between cancer and normal tissues in multiple cancers. Consistent with transcription levels, ADAR protein levels are significantly elevated in a variety of cancers (Figure 1D). Finally, to avoid bias in the analysis from a single database, we also collected independent datasets from multiple cancers in the GEO database. The results showed that ADAR expression was upregulated in most tumors except ACC, COAD, and PRAD, which was basically consistent with the analysis results from the TCGA database (Figure 1E).

### Effect of ADAR on clinical manifestations and prognosis of pan-cancer

Recognizing that ADAR expression is upregulated in cancer, we further explored its potential role in cancer malignancy. A boxplot showed that ADAR expression was positively correlated with a higher pathological grade in BLCA, CESC, LIHC, UCEC, and PAAD, with the highest confidence in BLCA (AUC = 0.732) (Supplementary Figure 1).

Cox hazard regression forest plots showed that high ADAR expression was associated with poor OS in ACC (HR = 2.73053, *P* = 0.0123), KIRP (HR = 8.65333, *P* = 0.0420), and LGG (HR = 1.85562, *P* = 0.0014) (Supplementary Figure 2A). Risk profiles and survival analyses confirm this conclusion. High AUC values indicate high reliability of the prediction (Supplementary Figure 2B–2D).

High expression of ADAR was significantly associated with poor DSS in ACC (HR = 2.80207, *P* = 0.0141), KIRP (HR = 2.60391, *P* = 0.0222), and LGG (HR = 1.76521, *P* = 0.0052) (Supplementary Figure 3A). This conclusion was further tested by risk distribution analysis, survival analysis, and ROC curve analysis (Supplementary Figure 3B–3D). In addition, Cox regression analysis was performed to investigate the impact of ADAR on pan-cancer PFS and DFS, and the results proved that ADAR was associated with worse PFS in ACC (HR = 2.96237, *P* = 0.0011), KICH (HR = 5.18454, *P* = 0.0355), KIRO (HR = 1.77391, *P* = 0.0364), LGG (HR = 1.33619, *P* = 0.0486) and UCEC (HR = 1.46373, *P* = 0.0357) and predicted better PFS in KIRC (HR = 0.70511, *P* = 0.0301). Similarly, high ADAR expression predicted poor DFS in CESC (HR = 2.41896, *P* = 0.0378) and KIRP (HR = 4.84632, *P* = 0.0015) but was positively associated with better DFS in LGG (HR = 0.3786, *P* = 0.0386) (Supplementary Figure 4A, 4B).

### Mutational landscape of ADAR in pan-cancer

**Figure 2:**
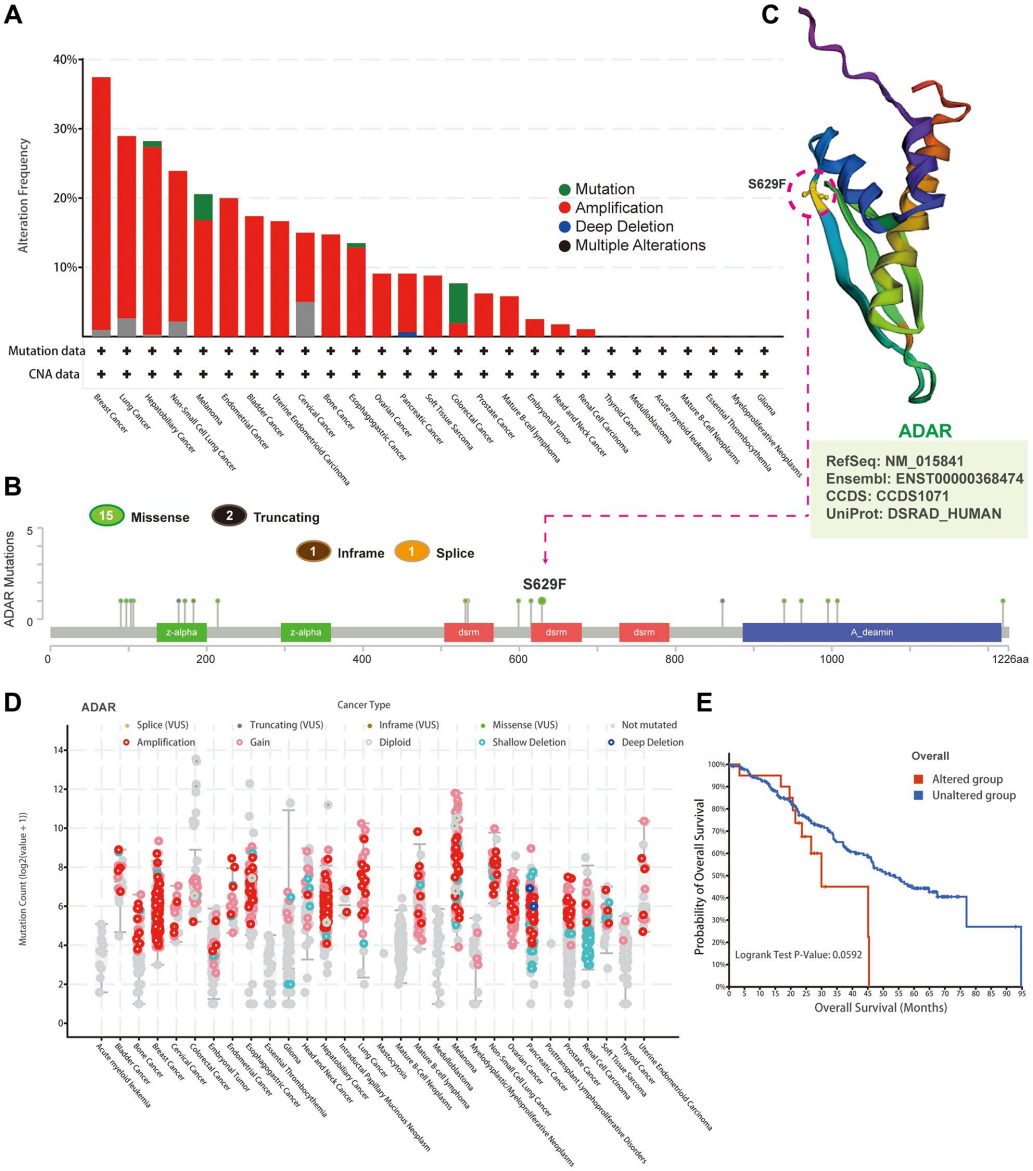
**Mutational landscape of ADAR in pan-cancer.** (**A**) The genetic alteration status of ADAR in pan-cancer through the cBioPortal database. (**B**) The types, loci, and case information of ADAR genetic alterations. (**C**) The 3D structure shows the S629F mutation site on ADAR protein. (**D**) A dot plot of ADAR gene alterations in pan-cancer. (**E**) Survival analysis between the altered and unaltered groups.

We observed the genetic alteration status of ADAR across cancers through the cBioPortal database. ADAR gained the highest alteration frequency in breast cancer and was dominated by amplification. In endometrial cancer, bladder cancer and uterine endometrioid carcinoma, almost all ADAR gene alterations were amplification (Figure 2A). The types, loci, and case information of ADAR genetic alterations are shown in Figure 2B. We found missense mutations represented by the S629F mutation to be the predominant type of genetic alteration in ADAR. The 3D structure also shows the S629F mutation site on the ADAR protein (Figure 2C). Subsequently, we generated a dot plot of ADAR gene alterations across cancers. Most cancers were dominated by amplification (Figure 2D). However, there was no significant difference in survival analysis between the altered and unaltered groups (Figure 2E).

### Interaction network of ADAR and enrichment analysis of related genes

**Figure 3:**
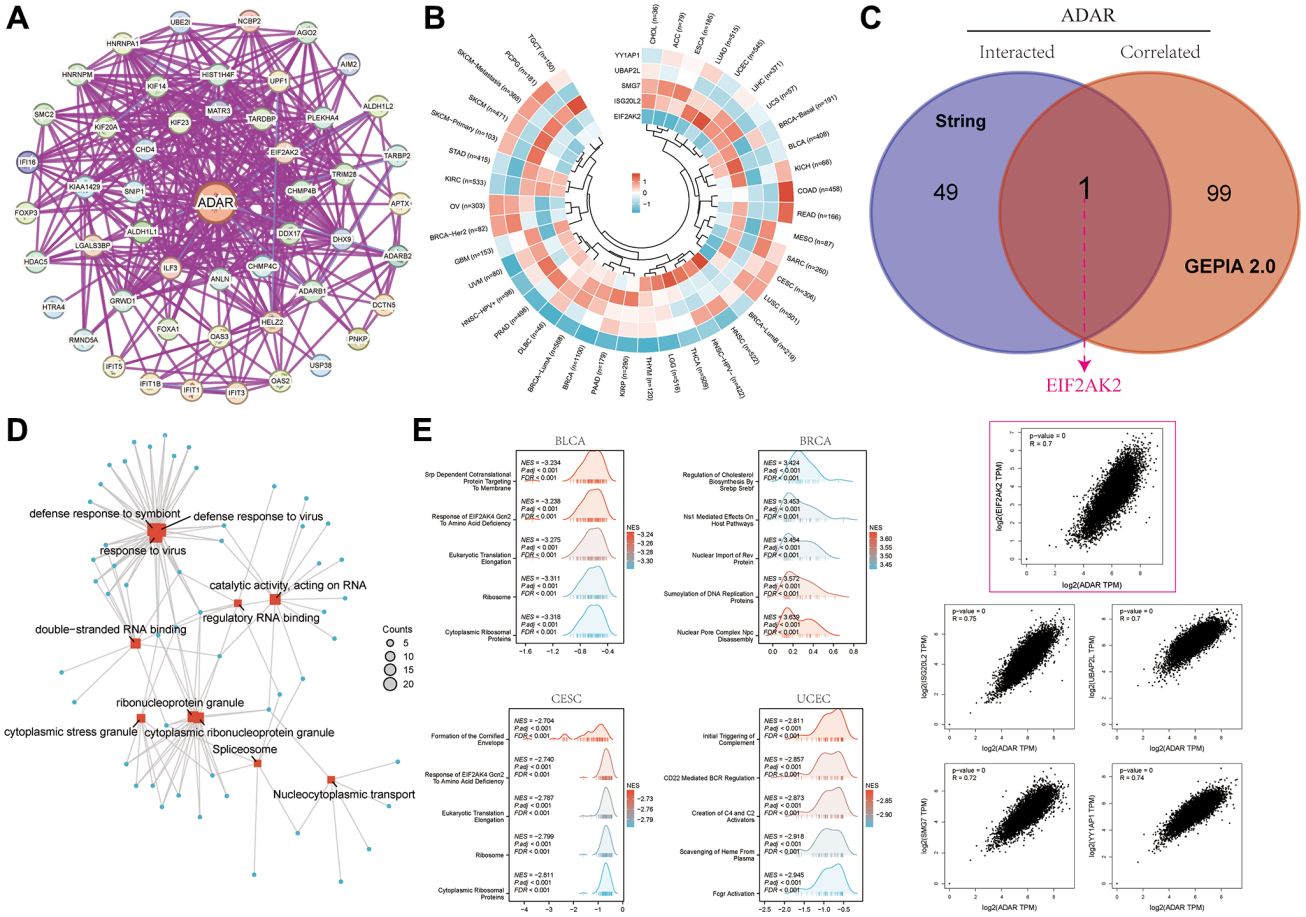
**Interaction network of ADAR and enrichment analysis of related genes.** (**A**) 50 experimentally verified ADAR interacting genes obtained from the String database. (**B**) A heat map of the specific associations of the five most highly correlated ADAR genes (EIF2AK2, ISG20L2, SMG7, UBAP2L, and YY1AP1) in pan-cancer. (**C**) EIF2AK2 was the most critical gene. We also presented correlations for EIF2AK2 and four additional significantly associated genes. (**D**) GO and KEGG enrichment analyses for all ADAR-related genes. (**E**) GSEA enrichment analysis revealed the molecular pathways of ADAR-related genes.

Fifty experimentally verified ADAR-interacting genes were obtained from the String database and are presented in Figure 3A. We subsequently obtained a list of the top 100 ADAR-related genes from the GEPIA 2.0 website (Supplementary Table 1). Figure 3B presents a heatmap of the specific associations of the five most highly correlated ADAR genes (EIF2AK2, ISG20L2, SMG7, UBAP2L, and YY1AP1) in pan-cancer. To obtain the genes most associated with ADAR, we performed intersection analysis of these two gene sets. The Venn map showed that EIF2AK2 was the most critical gene. We also presented correlations for EIF2AK2 and four additional significantly associated genes (Figure 3C). Subsequently, we performed GO and KEGG enrichment analyses for all ADAR-related genes. Among them, response to virus and RNA binding were the main enrichment functions (Figure 3D). GSEA revealed the molecular pathways of ADAR-related genes enriched in BLCA, BRCA, CESC, and UCEC, with the response of EIF2AK4 Gcn2 to amino acid deficiency, eukaryotic translation elongation, ribosomes, and cytoplasmic ribosomal proteins significantly enriched in both BLCA and CESC (Figure 3E).

### Significant correlation between ADAR expression and immune infiltration of cancers

**Figure 4:**
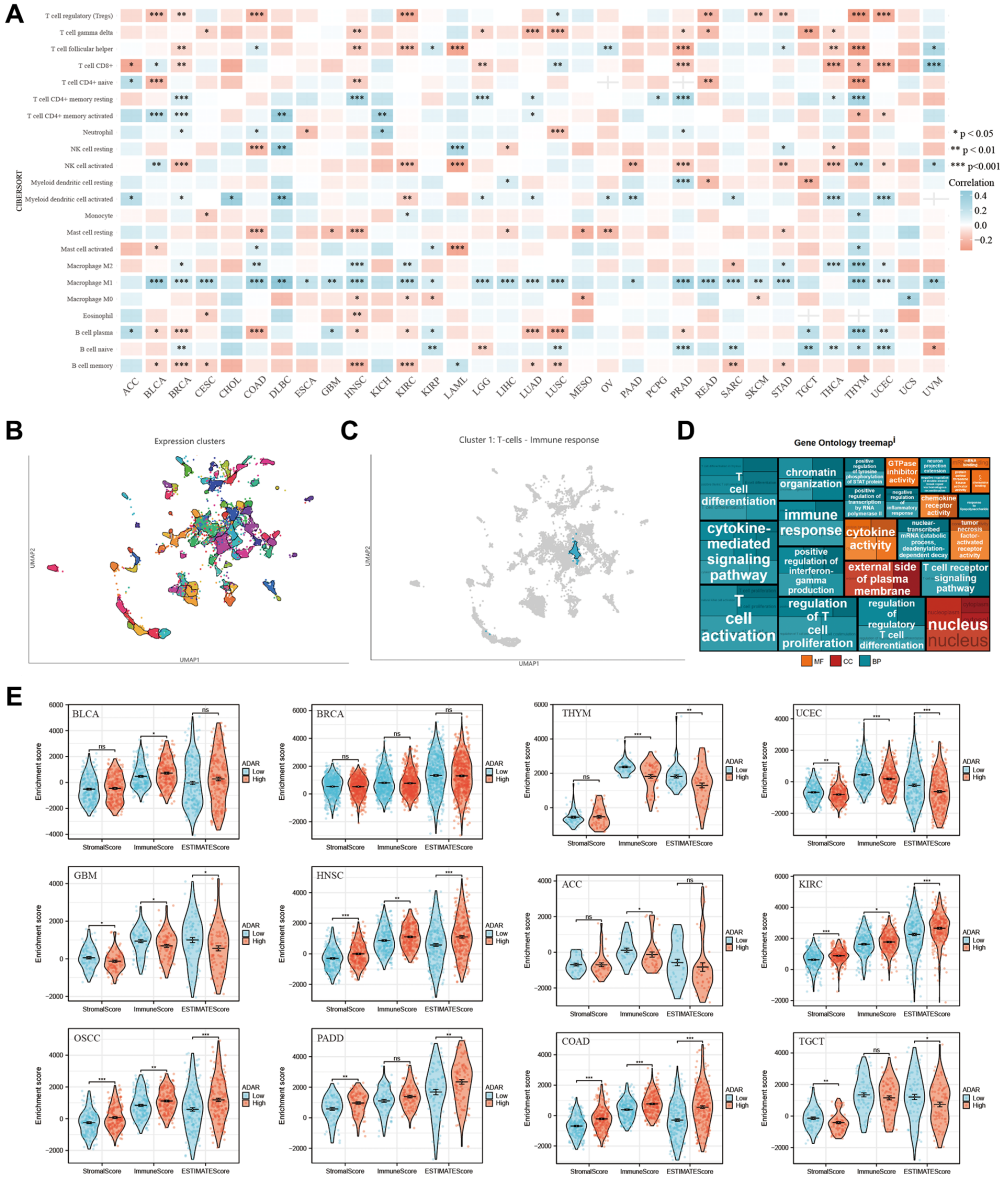
**Correlation between ADAR Expression and immune infiltration of cancers.** (**A**) ADAR was correlated with the number of immune cells in most tumors. (**B**, **C**) In the HPA single-cell dataset, correlation analysis between ADAR expression and immune cell clustering revealed that ADAR is a part of cluster-1 (T cells—immune response), with high annotation reliability. (**D**) Gene ontology treemap describes that ADAR is significantly correlated with immune response and T cell activation, differentiation, and proliferation. (**E**) Negative correlations were observed in ACC, GBM, TGCT, THYM, and UCEC.

Analysis of the correlation between ADAR expression and immune infiltration of cancer revealed that ADAR was correlated with the number of immune cells in most tumors, with the most significant positive correlation with the infiltration of M1 macrophages (Figure 4A). Furthermore, in the HPA single-cell dataset, correlation analysis between ADAR expression and immune cell clustering revealed that ADAR is a part of cluster-1 (T cells—immune response), with high annotation reliability (Figure 4B, 4C, Supplementary Table 2). Gene ontology treemap describes that ADAR is significantly correlated with immune response and T cell activation, differentiation, and proliferation (Figure 4D). It is worth noting that ADAR and Treg cells showed obvious negative correlations in a variety of cancers, such as BLCA, BRCA, COAD, KIRP, READ, SKCM, STAD, THYM, and UCEC. A positive correlation was only shown in LUSC. In BLCA, LUSC, and UVM, ADAR and CD8+ T cells showed a strong positive correlation. However, strong inverse associations were observed in ACC, BRCA, LGG, PRAD, TICH, THYM, and UCEC. Meanwhile, ADAR was found to be positively correlated with the immune score and stromal score of some cancers, such as BLCA, COAD, HNSC, KIRC, OSCC, and PDAC. Negative correlations were observed in ACC, GBM, TGCT, THYM, and UCEC (Figure 4E). This indicates the complexity of the regulatory mechanisms of the tumor immune microenvironment and the functional diversity of ADAR.

### Involvement of ADAR in the tumor immune response

**Figure 5:**
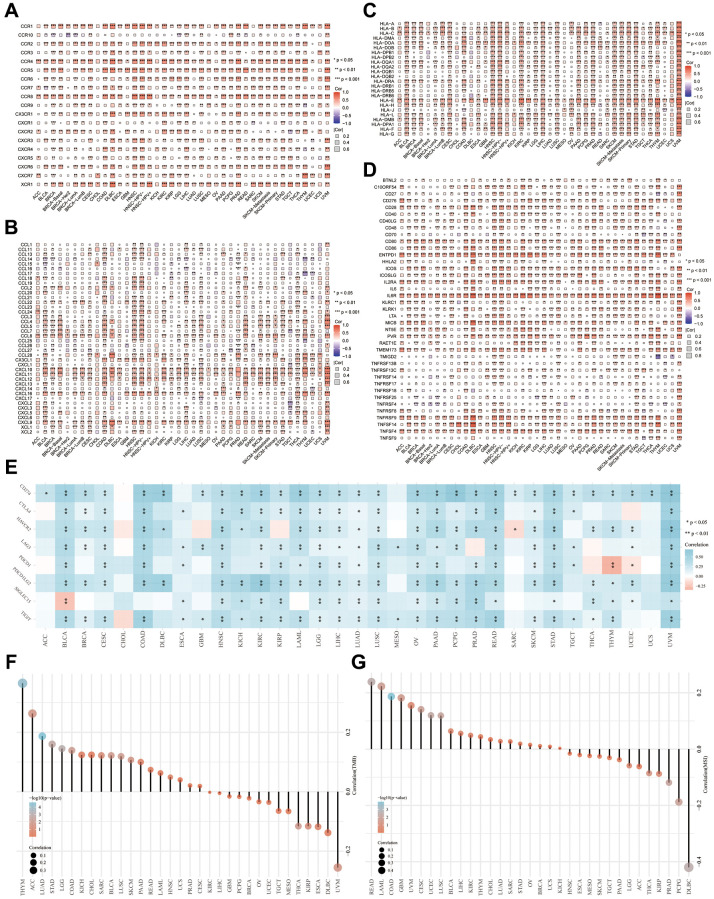
**Involvement of ADAR in tumor immune response.** (**A**–**D**) ADAR is positively correlated with the expression of chemokines, chemokine receptors, HLAs, and tumor necrosis factors (TNF) in most cancers. (**E**) Correlation analysis between ADAR and ICIs. (**F**) ADAR and TMB achieved high positive correlation in THYM, ACC, LUAD, STAD, LGG, COAD, KICH, CHOL, SARC, and BLCA. (**G**) ADAR was significantly associated with MSI in READ, LAML, COAD, GBM, UVM, CESC, UCEC, LUSC, BLCA, and LIHC.

In cancer, chemokines play a key role in the pattern of immune cell migration into tumors [17]. Human leukocyte antigen (HLA), also known as major histocompatibility complex (MHC), is a protein molecule that exists on the surface of antigen-presenting cells and is responsible for antigen presentation [18]. HLA has been reported to predict the response and prognosis of immunotherapy [19]. Our results show that ADAR is positively correlated with the expression of chemokines, chemokine receptors, HLAs, and tumor necrosis factors (TNFs) in most cancers (Figure 5A–5D). Among them, the most significant genes were CCR1, CCR4, CCR8, XCR1, CXCL10, CXCL11, HLA-E, ENTPD1, IL6R, etc.

ICIs (especially PD-1), TMB, and MSI are now being used as predictive markers of response to immunotherapy [20–22]. Correlation analysis between ADAR and ICI expression showed a highly positive correlation in most cancers (Figure 5E). These findings, especially for BLCA, COAD, and UVM, suggest that ADAR is involved in the regulation of tumor immune responses through the regulation of ICIs. ADAR and TMB achieved a high positive correlation in THYM, ACC, LUAD, STAD, LGG, COAD, KICH, CHOL, SARC, and BLCA (Figure 5F). ADAR was significantly associated with MSI in READ, LAML, COAD, GBM, UVM, CESC, UCEC, LUSC, BLCA, and LIHC (Figure 5G).

### Potential functions and molecular pathways of ADAR in BLCA

From the results of the above analysis, we can see that BLCA has the most significant upregulation of ADAR with the highest AUC value. Although ADAR appears to have no effect on BLCA survival, the effect of ADAR on the degree of BLCA malignancy had the highest confidence. Therefore, it is necessary to further explore the potential role of ADAR in BLCA, especially its potential value in BLCA immunotherapy.

**Figure 6:**
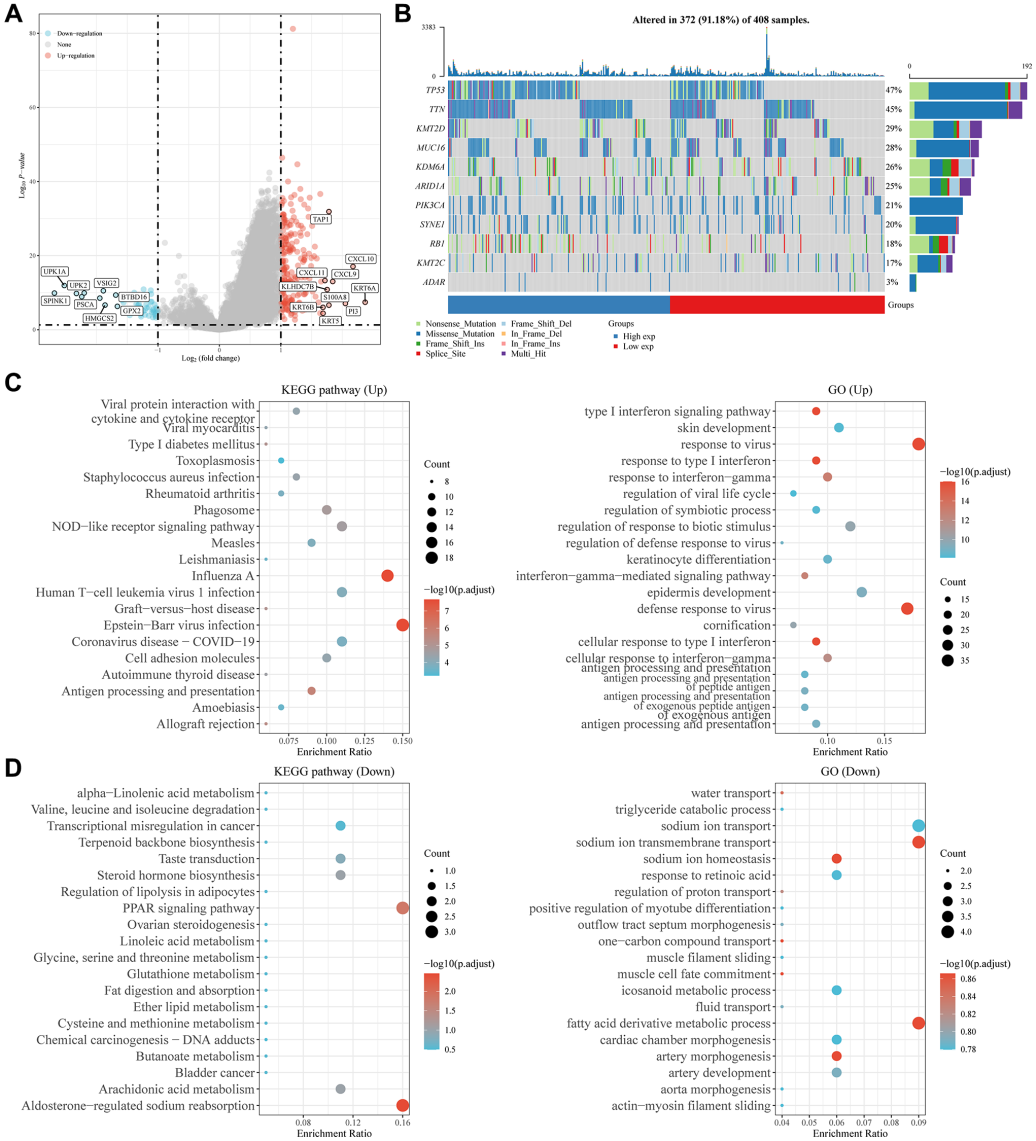
**Potential functions and molecular pathways of ADAR in BLCA.** (**A**) Differential analysis between the ADAR high-expression group and the ADAR low-expression group. (**B**) High ADAR expression predicts a higher frequency of gene mutations. (**C**, **D**) GO and KEGG enrichment analysis of differentially expressed genes.

Table 1 presents a multivariable Cox regression of ADAR and clinical features of BLCA in TCGA. We can conclude that ADAR is associated with a higher immunotherapy response and predicts a more malignant grade of BLCA (*P* = 0.014). We performed differential analysis between the ADAR high-expression group and the ADAR low-expression group. Fifty differentially expressed genes with low expression and 228 differentially expressed genes with high expression were obtained (Supplementary Table 3). The KRT and CXCL gene families showed significant positive correlations with ADAR (Figure 6A). The gene mutation landscape of the ADAR high- and low-expression groups indicated that the high ADAR expression group predicted a higher frequency of gene mutations (Figure 6B). GO and KEGG enrichment analyses of differentially expressed genes showed that the highly expressed ADAR group was significantly enriched in Epstein Barr virus infection and influenza A pathways; it was also related to the defense response to virus and response to virus (Figure 6C). The ADAR low expression group showed enrichment of the PPAR signaling pathway, aldosterone-regulated sodium reabsorption, sodium ion transmembrane transport, fatty acid derivative metabolic process and other pathways and functions (Figure 6D).

**Table 1:** Multivariable Cox regression of ADAR and clinical features of BLCA in TCGA.

**Characteristics**	**Low expression of ADAR**	**High expression of ADAR**	***P*-value**
*n*	206	206	
Age, *n* (%)			0.112
≤70	108 (26.2%)	124 (30.1%)	
>70	98 (23.8%)	82 (19.9%)	
Gender, *n* (%)			0.502
Female	51 (12.4%)	57 (13.8%)	
Male	155 (37.6%)	149 (36.2%)	
Primary therapy outcome, *n* (%)			0.032
PD	29 (8.2%)	41 (11.5%)	
SD	10 (2.8%)	20 (5.6%)	
PR	15 (4.2%)	7 (2%)	
CR	122 (34.4%)	111 (31.3%)	
Histologic grade, *n* (%)			0.014
Low grade	16 (3.9%)	5 (1.2%)	
High grade	189 (46.2%)	199 (48.7%)	
Pathologic stage, *n* (%)			0.560
Stage I	1 (0.2%)	3 (0.7%)	
Stage II	65 (15.9%)	64 (15.6%)	
Stage III	67 (16.3%)	75 (18.3%)	
Stage IV	72 (17.6%)	63 (15.4%)	
Smoker, *n* (%)			0.052
No	63 (15.8%)	46 (11.5%)	
Yes	136 (34.1%)	154 (38.6%)	
OS event, *n* (%)			0.321
Alive	120 (29.1%)	110 (26.7%)	
Dead	86 (20.9%)	96 (23.3%)	

### Great value of ADAR in the immunotherapy of BLCA

**Figure 7:**
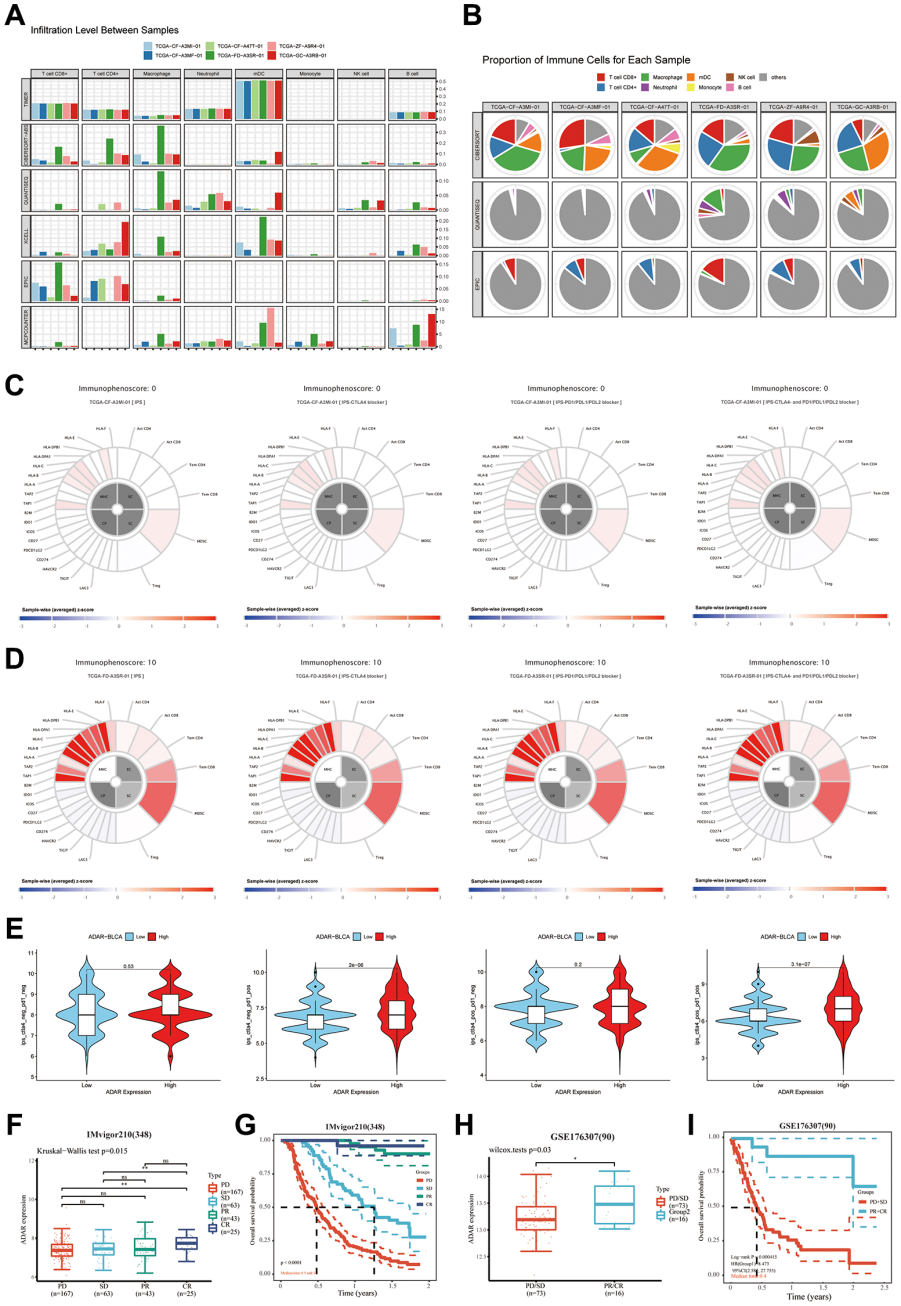
**The great value of ADAR in the immunotherapy of BLCA.** (**A**) The ADAR high expression group had a higher number of immune cells in the tumor microenvironment. (**B**) CD4+T cells accounted for a greater proportion of patients with high expression of ADAR. (**C**, **D**) ADAR contribute to higher immunotherapy responses. (**E**) IPS scores on all TCGA-BLCA samples reconfirmed the conclusion that high ADAR expression predicted a better immunotherapy response. (**F**) The difference boxplot from the immunotherapy cohort IMvigor210 (*n* = 348). (**G**) Survival analysis showed that patients in PR and CR groups had significantly better survival expectations than those in PD and SD groups. (**H**, **I**) The difference boxplot and survival analysis from the immunotherapy cohort GSE176307 (*n* = 90).

Tumor immunotherapy has become the focus of discussion among oncologists. Therefore, it is important to explore the potential immunotherapy response markers of BLCA. We explored the immune cell infiltration landscape of cancer samples with the highest ADAR expression (TCGA-FD-A3SR-01, TCGA−FD−A3SR−01, TCGA−GC−A3RB−01) and the lowest ADAR expression (TCGA-CF-A3MI-01, TCGA−CF−A47T−01, and TCGA−ZF−A9R4−01) in TCGA-BLCA using the Timer 2.0 database. In general, the ADAR high expression group had a higher number of immune cells in the tumor microenvironment (Figure 7A). Among them, CD4+ T cells accounted for a greater proportion of patients with high expression of ADAR (Figure 7B). Subsequently, separate analyses targeting the responsiveness of samples with the highest and lowest ADAR expression to immunotherapy revealed that ADAR contributed to higher immunotherapy responses (Figure 7C, 7D). This is consistent with the above conclusion that ADAR is positively correlated with PD-1, CTLA4, and PD-L1. Subsequently, IPSs on all TCGA-BLCA samples reconfirmed that ADAR does not significantly reflect the efficacy of CTLA4-independent immunotherapy. However, when PD1 immunotherapy was used alone or in combination, the high ADAR expression group showed a higher therapeutic benefit. This suggests that ADAR can be used as a superior response marker for PD1 immunotherapy (the recommended immunotherapy strategy for patients with muscle-invasive and metastatic BLCA [23] in patients with BLCA (Figure 7E). The difference boxplot from the immunotherapy cohort IMvigor210 (*n* = 348) showed that the expression of ADAR in the complete response (CR) group was significantly higher than that in the progressive disease (PD) and stable disease (SD) groups but not significantly different from that in the partial response (PR) group (Figure 7F). Survival analysis showed that patients in the PR and CR groups had significantly better survival expectations than those in the PD and SD groups (Figure 7G). The same results were obtained in another immunotherapy cohort, GSE176307 (*n* = 90) (Figure 7H, 7I).

### Validation of ADAR expression and function in BLCA

**Figure 8:**
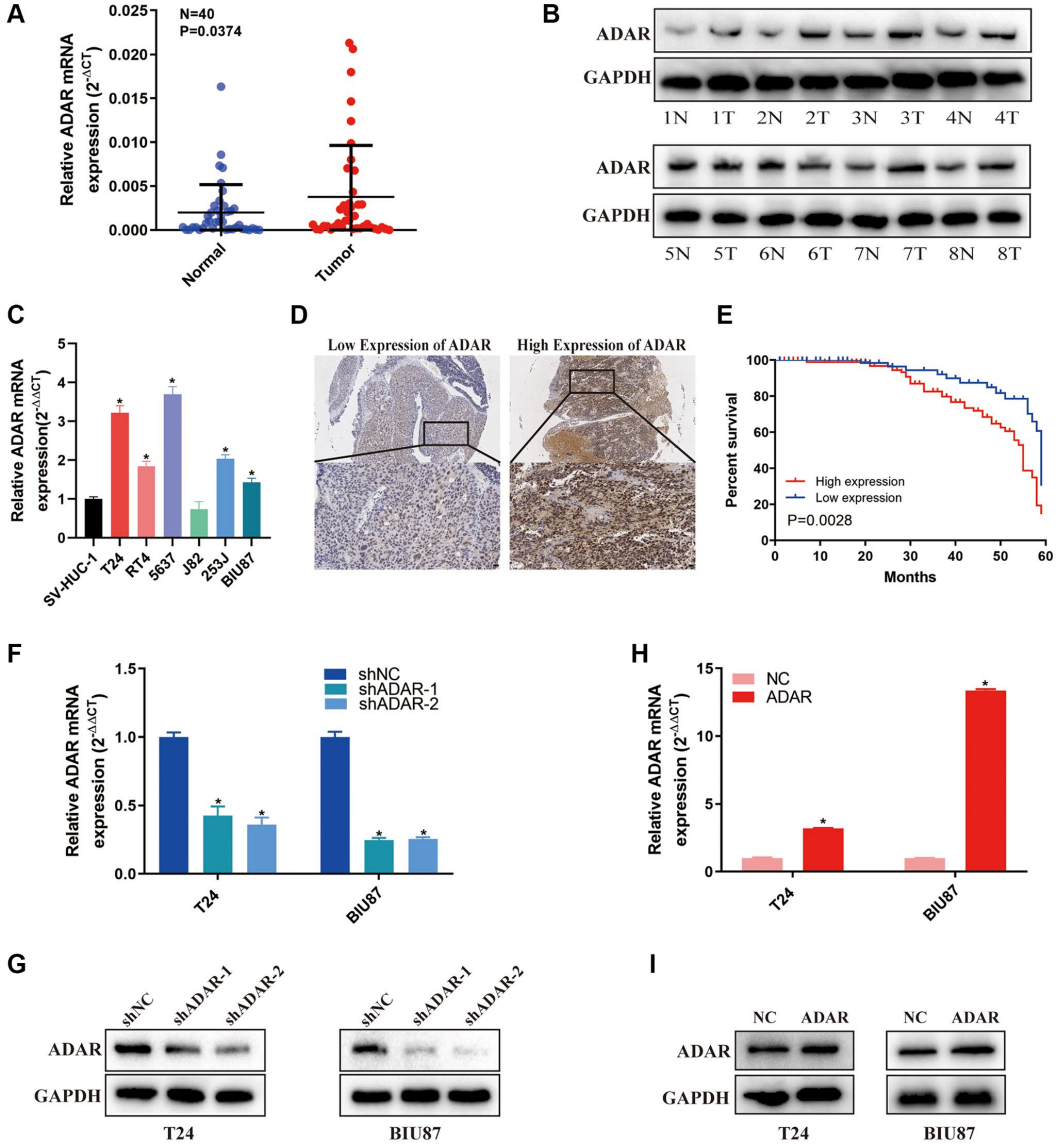
**ADAR was up-regulated in bladder cancer tissues and served as a prognostic factor in bladder cancer.** (**A**) Relative expression of ADAR mRNA in the 40 pairs of bladder cancer tissues and matched adjacent normal tissues quantified by qRT-PCR. ADAR was up-regulated in bladder cancer tissues compared with that in adjacent normal tissues. (**B**) The expression of ADAR protein in 8 pair bladder cancer tissues (T) and adjacent normal tissues (N) by western blot were shown. (**C**) Relative expression of ADAR in bladder cancer cell lines and normal bladder epithelial cell line SV-HUC-1 by qRT-PCR. Data represent the mean ± SD from three independent experiments, ^*^P < 0.05. (**D**) IHC analysis of ADAR in bladder cancer tissue at 100× and 400× magnification. (**E**) Kaplan-Meier survival curves of overall survival in 180 bladder cancer patients based on ADAR by IHC staining. The log-rank test was used to compare differences between two groups (*P* = 0.0028). (**F**, **G**) Validation of the knockdown efficacy of ADAR in T24 and BIU87 cell lines by qRT-PCR and western blot. Data represent the mean ± SD from three independent experiments, ^*^P < 0.05. (**H**, **I**) The overexpression efficacy of ADAR in T24 and BIU87 cell lines by qRT-PCR and western blot. Data represent the mean ± SD from three independent experiments, ^*^P < 0.05.

By bioinformatics analysis, we identified the important role of ADAR in BLCA, especially as a biomarker for the progression and response to immunotherapy. For more reliable verification, we further confirmed the expression and function of ADAR in BLCA by experiments. We examined the expression of ADAR in bladder cancer tissues (*n* = 40) and matched adjacent normal tissues (*n* = 40) by qRT-PCR. ADAR was significantly upregulated in bladder cancer tissues, which was consistent with the bioinformatics analysis results (Figure 8A). The protein levels of ADAR in 8 pairs of bladder cancer tissues (T) and adjacent normal tissues (N) were detected by western blotting, and the results were consistent with those obtained by qRT-PCR (Figure 8B). ADAR was also upregulated in five bladder cancer cell lines (T24, RT4, 5637, 253J and BIU87) compared with SVHUC-1 (human ureteral epithelial immortalized cell line, as the normal urothelial cell line) (Figure 8C). We subsequently performed IHC analysis of bladder cancer tissues from 180 patients, and Figure 8D shows pictures of the results with high and low ADAR expression. The survival analysis revealed that ADAR is a risk factor for BLCA and predicts poor survival expectations (Figure 8E). These data suggest that ADAR is upregulated in BLCA and is detrimental for survival. Table 2 presents correlations between the expression of ADAR and clinicopathological features in the 180 BLCA patients. We can conclude that the high expression of ADAR is positively correlated with the histological grade (<0.001) and Tumor size (*P* = 0.017) of bladder cancer. In addition, the higher the ADAR expression, the larger the predicted tumor (*P* = 0.0079). To further investigate the functional effects of ADAR on bladder cancer cells, we selected high-grade bladder cancer cell line T24 and low-grade bladder cancer cell line BIU87 to conduct experimental verification. ADAR was knocked down/overexpressed in T24 cells and BIU87 cells. Figure 8F–8I shows knockdown/overexpression efficiency of ADAR.

**Table 2:** Correlations between the expression of ADAR and clinicopathological features in BLCA patients.

**Characteristics**	**Case**	**ADAR**		***P*-value**
		**Low**	**High**	
All cases, *n* (%)	180	75 (41.7)	105 (58.3)	
Age (years), *n* (%)				0.803
<65	39	16 (41.0)	23 (59.0)	
≥65	141	61 (43.3)	80 (56.7)	
Gender, *n* (%)				0.772
Male	133	59 (44.4)	74 (55.6)	
Female	47	22 (46.8)	25 (53.2)	
TNM stage, *n* (%)				0.938
pTa-pT1	104	35 (33.7)	69 (66.3)	
pT2-pT4	76	26 (34.2)	50 (65.8)	
Histological grade				<0.001^*^
Low	117	72 (61.5)	45 (38.5)	
High	63	17 (27.0)	46 (73.0)	
Tumor size (cm)				0.017^*^
<3	103	56 (54.4)	47 (45.6)	
≥3	77	28 (36.4)	49 (63.6)	

^*^P < 0.05.

**Figure 9:**
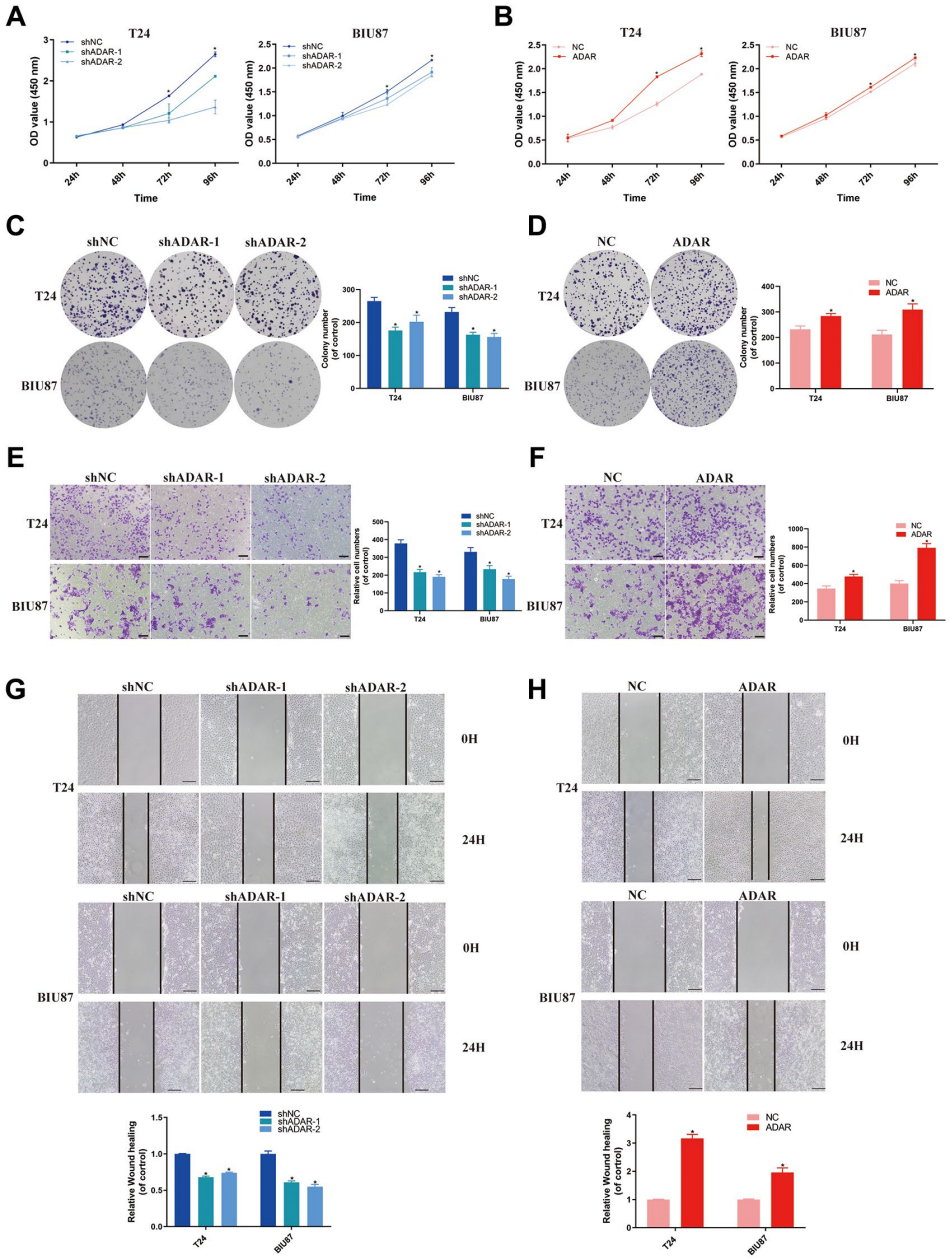
**ADAR promoted bladder cancer cell proliferation, invasion and migration *in vitro*.** (**A**, **B**) Cell proliferation assessed by CCK8 assays. Knockdown of ADAR inhibited cell proliferation in T24 and BIU87 cells. Overexpression of ADAR promoted cell proliferation in T24 and BIU87 cells. Data represent the mean ± SD from three independent experiments, ^*^P < 0.05. (**C**, **D**) Colony formation assay showed that knockdown of ADAR significantly decreased the cloning number of T24 and BIU87 cells compared with control group, while ADAR overexpression significantly increased the cloning number of T24 and BIU87 cells. Data represent the mean ± SD from three independent experiments, ^*^P < 0.05. (**E**, **F**) Invasion assay (Transwell) in T24 and BIU87 cell lines were measured. The results were expressed of crossing cells number per field compared with respective control. Magnification: 100×. Data represent the mean ± SD from three independent experiments, ^*^P < 0.05. (**G**, **H**) Migration assay (Wound healing) in T24 and BIU87 cell lines were measured. Knockdown of ADAR inhibited cell migration in T24 and BIU87 cells after 24 hours. Overexpression of ADAR promoted cell migration in T24 and BIU87 cells after 24 hours. Data represent the mean ± SD from three independent experiments, ^*^P < 0.05.

CCK-8 assays showed that ADAR knockdown significantly inhibited the proliferation of bladder cancer cells (Figure 9A). However, overexpression of ADAR had the opposite effect (Figure 9B). Colony assays showed that inhibition of ADAR significantly reduced the clone numbers of T24 cells and BIU87 cells (Figure 9C). However, the opposite results were observed when ADAR was upregulated (Figure 9D). These results suggest that ADAR can promote the proliferation of bladder cancer cells. In addition, invasion assays (Transwell) were performed in T24 and BIU87 cell lines. Knockdown of ADAR inhibited the invasion of T24 and BIU87 cells (Figure 9E). Overexpression of ADAR promoted the invasion of T24 and BIU87 cells (Figure 9F). Finally, we performed a migration assay (wound healing), and the results showed that downregulation of ADAR inhibited the migration ability of bladder cancer cells, while upregulation of ADAR achieved the opposite effect (Figure 9G, 9H). Taken together, these *in vitro* experiments demonstrated that ADAR plays a key role in promoting the proliferation, migration, and invasion of bladder cancer cells, thus promoting the progression of BLCA.

## DISCUSSION

Cancer seriously affects human health and is now the second leading cause of death worldwide. Tumor therapy mainly includes traditional surgical therapy, radiotherapy, chemotherapy and new therapies such as targeted therapy and immunotherapy, which have developed rapidly in recent years. In addition, the combination of next-generation sequencing and advanced computational data analysis methods has revolutionized our understanding of the genomic basis of cancer development and progression [24]. Pan-cancer bioinformatics analysis of these data allows us to understand the major pathological functions and mechanisms of a key gene and explore its role and potential clinical value in tumorigenesis, progression, and treatment response in a specific cancer. ADAR has been shown to be a central protein involved in RNA editing [25]. However, the potential oncogenic effects of ADAR and its value in tumor therapy deserve further investigation and exploration. In this study, we systematically elucidated the integrated landscape of ADAR in cancer. By combining different bioinformatics platforms and datasets from different sources, we comprehensively analyzed the differential expression, prognostic significance, mutation landscape, gene interaction network, regulation of ADAR in the tumor microenvironment, and predictive value in cancer immunotherapy. We hope that this study will provide new insights into improving the role of ADAR in cancer and tumor immunotherapy.

Previous studies have reported that dysregulation of ADAR is closely related to carcinogenesis and malignant progression of tumors. For example, ADAR can promote the development of thyroid cancer through RNA editing of CDK13 [26]. Inhibition of ADAR expression can significantly inhibit the proliferation, invasion and migration of thyroid cancer cells, reflecting the strong carcinogenic effect of ADAR [27]. Based on these clues, we comprehensively explored the expression of ADAR across cancers. We found that ADAR mRNA and protein expression was significantly higher in most tumor tissues than in normal tissues. In addition, high expression of ADAR predicts a higher pathological grade. Furthermore, ADAR is associated with the survival of patients with a variety of cancers. For example, high expression of ADAR predicts poor overall survival in ACC, KICH, and LGG. Similarly, PFS, DSS, and DFS analyses showed that ADAR was an unfavorable factor for tumor patients. Therefore, it is of great value to explore ADAR for tumor prediction and diagnosis. 8-Azaadenosine is a potent ADAR1 inhibitor and an A-to-I editing inhibitor. It has been verified by experiments that 8-azaadenosine can inhibit the proliferation of thyroid cancer cells and suppress the progression and peritoneal metastasis of gastric cancer by inhibiting ADAR [27, 28]. The clinical value of 8-azaadenosine will be realized by its combination with existing cancer treatment drugs.

The activation or alteration of signaling pathways is fundamental to disease occurrence [29]. Oncogenes or tumor suppressor genes lead to the initiation or attenuation of cancer by affecting their downstream signaling pathways. Enrichment analysis showed that ADAR and its interacting genes were mainly involved in immune and tumor-related pathways such as response to viruses, spliceosome formation and regulatory RNA binding. This is consistent with previous research reports that ADAR-induced deamination of RNA is a significant source of mutant SARS-CoV-2 [30]. These results suggest that ADAR is closely related to cancer development and immunity.

Furthermore, we evaluated the pan-cancer immune infiltration landscape and found that ADAR was associated with infiltrating immune cells. The tumor immune microenvironment is mainly composed of tumor cells, stromal cells and immune cells. As one of the main components, immune cells play an important role in antitumor immunity and protumor immunity [31]. Oncogenes or tumor suppressor genes can participate in the reconstruction of the tumor immune microenvironment by regulating immune cells, thereby inhibiting or promoting antitumor immunity [32]. One study showed that the deletion of PTPN2 phosphatase in T cells promotes antitumor immunity and CAR T-cell efficacy in solid tumors [33]. Another study demonstrated that ADAR promotes T-cell migration to human melanoma cells [34]. In addition, ADAR has been shown to improve Treg cell function via the miR-21b/Foxp3 axis [35]. Our study showed that ADAR is associated with the activation of M1 macrophages. This evidence indicates the regulatory effect of ADAR on immune cells in the tumor microenvironment. We also revealed the relationship between ADAR and chemokines, chemokine receptors, and other immune-related factors. The complexity of the regulatory network between ADAR and immune microenvironment components suggests that the mechanism of ADAR is multifaceted. The application of immune checkpoint inhibitors (ICIs) has revolutionized the treatment of various cancers. However, despite success in some cancer patients, a significant proportion of patients do not respond to immune checkpoint inhibitors [36]. Our study found that ADAR was positively correlated with the expression of multiple immune checkpoints, which may indicate a favorable response to immunotherapy. TMB and MSI have become effective immunotherapy response markers in cancer. A high TMB means that more tumor neoantigens are exposed, so a high TMB predicts more effective immunotherapy. MSI presents as a DNA mismatch repair defect and is a marker of a good response to immunotherapy [21, 37]. Our study showed that in COAD and BLCA, ADAR was proportional to both TMB and MSI. This shows the clinical value of ADAR as a response marker in the immunotherapy of these two cancers.

ADAR plays an important role in the disease progression and immunotherapy of bladder cancer. However, the specific performance of ADAR in the immunotherapy response of bladder cancer has not yet been reported by urologists, so we chose BLCA to further analyze ADAR. We first investigated the specific molecular pathways by which ADAR may be involved in bladder cancer by bioinformatics analysis. Among them, Epstein Barr virus infection and influenza A pathways were significantly enriched. ADAR has also been found to be associated with the immune response to viruses. In addition, the TCGA-BLCA samples with the highest expression of ADAD showed significantly increased infiltration levels of immune cells such as CD4+ cells, macrophages and DCs. Moreover, the IPS showed that high expression of ADAR predicted a better response to PD-1 blockade in BLCA. This is consistent with the published results of two immunotherapy cohorts (GSE176307 and IMvigor210) of BLCA. We therefore hypothesized that RRM2 may play a role as an immunotherapy predictive marker in BLCA. Furthermore, in our collected clinical samples, ADAR was significantly upregulated in bladder cancer tissues and was associated with poor patient survival. Moreover, *in vitro* experiments showed that ADAR effectively promoted the proliferation, migration, and invasion of bladder cancer cells. Taken together, these results suggest that ADAR is a key regulator and plays an important role in BLCA development, progression, and immunotherapy. With further exploration, ADAR may become a promising therapeutic target for BLCA.

## CONCLUSION

In summary, we demonstrated that ADAR is ubiquitously expressed across cancers and is associated with poor clinical outcomes in most cancers. In addition, we illustrated the complex relationship between ADAR and the tumor immune microenvironment and propose the hypothesis that ADAR may be involved in the regulation of the tumor immunotherapy response. Finally, we propose the important role of ADAR in BLCA, which can be used as a predictive marker for immunotherapy response and as a therapeutic target.

## Supplementary Materials

Supplementary Figures

Supplementary Tables 1-2

Supplementary Table 3
