# Supplementary material for: Clinical significance, tumor immune landscape and immunotherapy responses of ADAR in pan-cancer and its association with proliferation and metastasis of bladder cancer
Source: Aging (Albany NY). 2023 Jul 6;15(13):6302–30. doi: 10.18632/aging.204853 (PMC10373965; doi:10.18632/aging.204853)
Supplement: Supplementary Figures [file aging-15-204853-s001.pdf]

SUPPLEMENTARY FIGURES

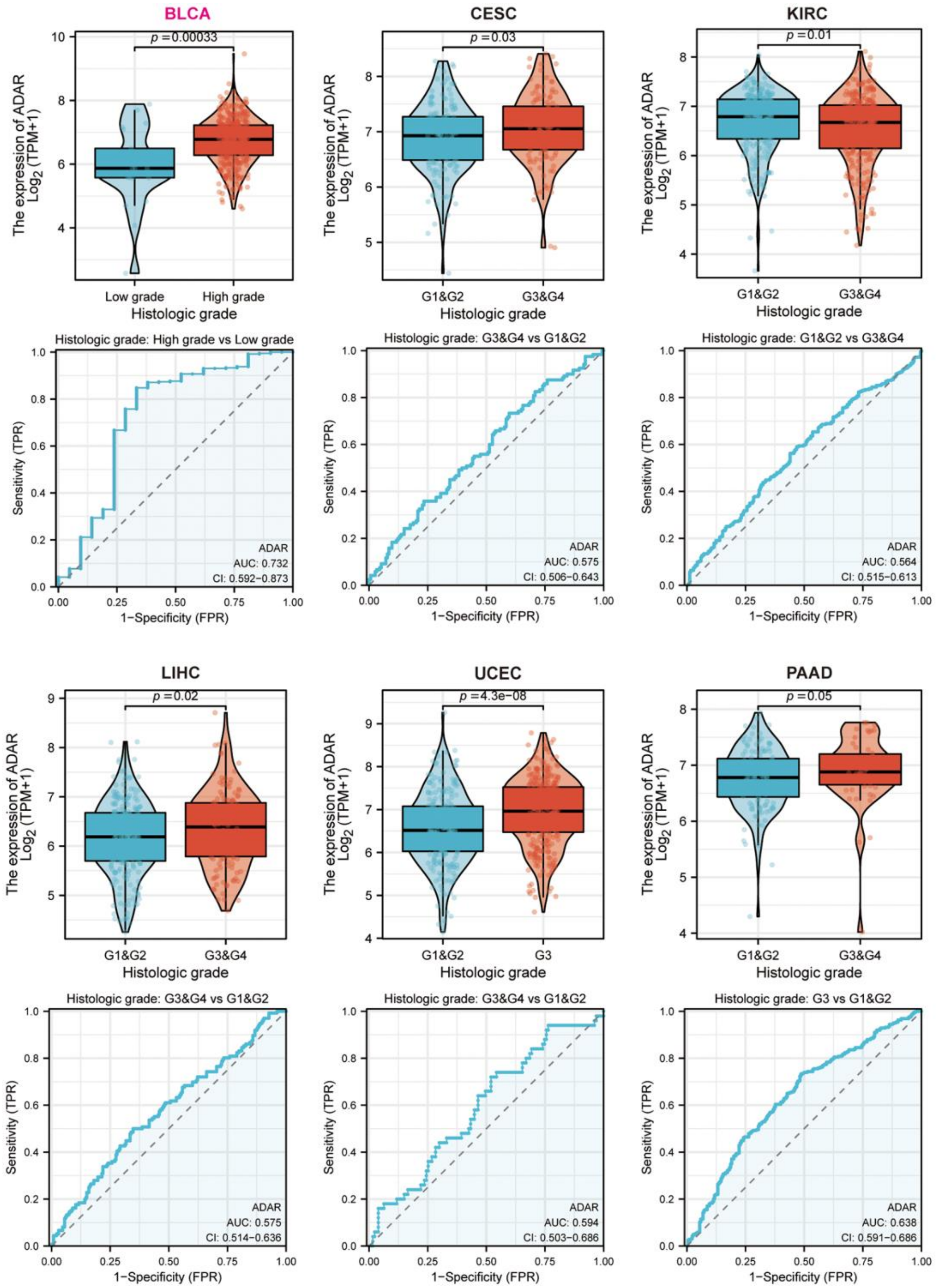

Supplementary Figure 1. ADAR expression was positively correlated with higher pathological grade in BLCA, CESC, LIHC, UCEC, and PAAD.

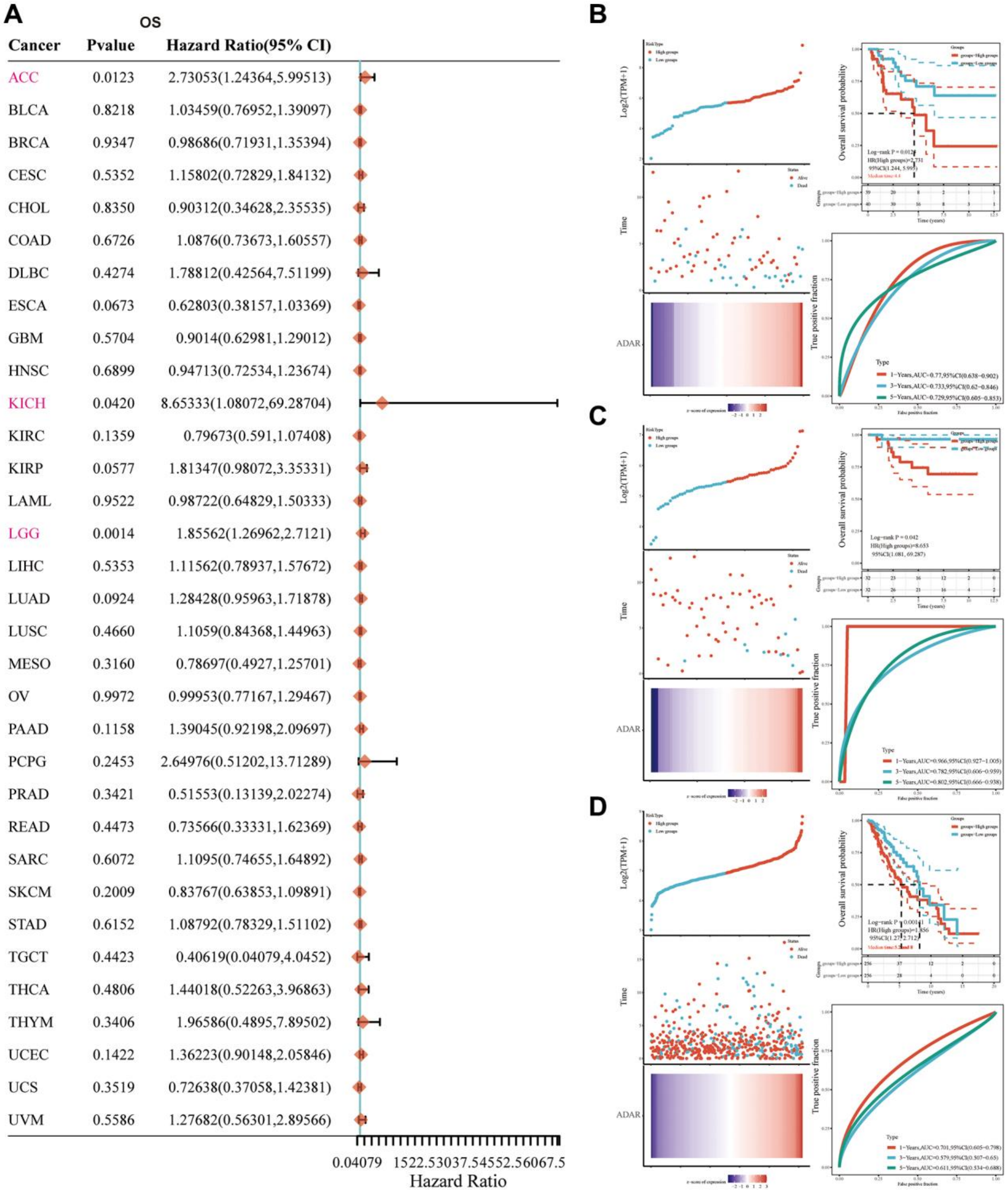

Supplementary Figure 2. ADAR expression with OS. (**A**) ADAR expression was associated with poor OS in ACC (HR = 2.73053, *P* = 0.0123), KIRP (HR = 8.65333, *P* = 0.0420), and LGG (HR = 1.85562, *P* = 0.0014). (**B**–**D**) Risk profiles and survival analyses of ADAR. High AUC values indicate high reliability of the prediction.

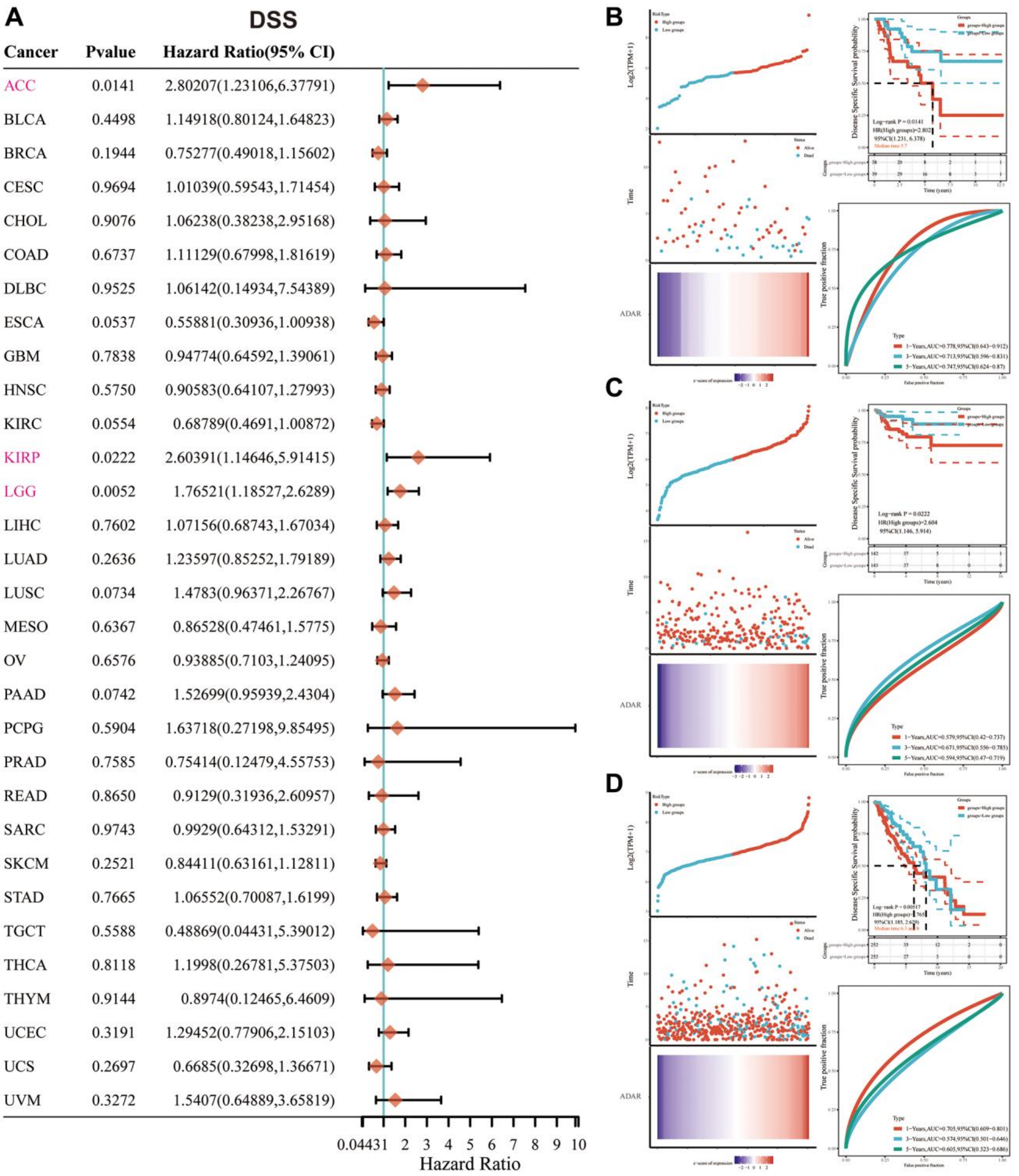

Supplementary Figure 3. ADAR expression with DSS. (**A**) ADAR was significantly associated with poor DSS in ACC (HR = 2.80207, *P* = 0.0141), KIRP (HR = 2.60391, *P* = 0.0222), and LGG (HR = 1.76521, *P* = 0.0052). (**B**–**D**) Risk profiles and survival analyses of ADAR. High AUC values indicate high reliability of the prediction.

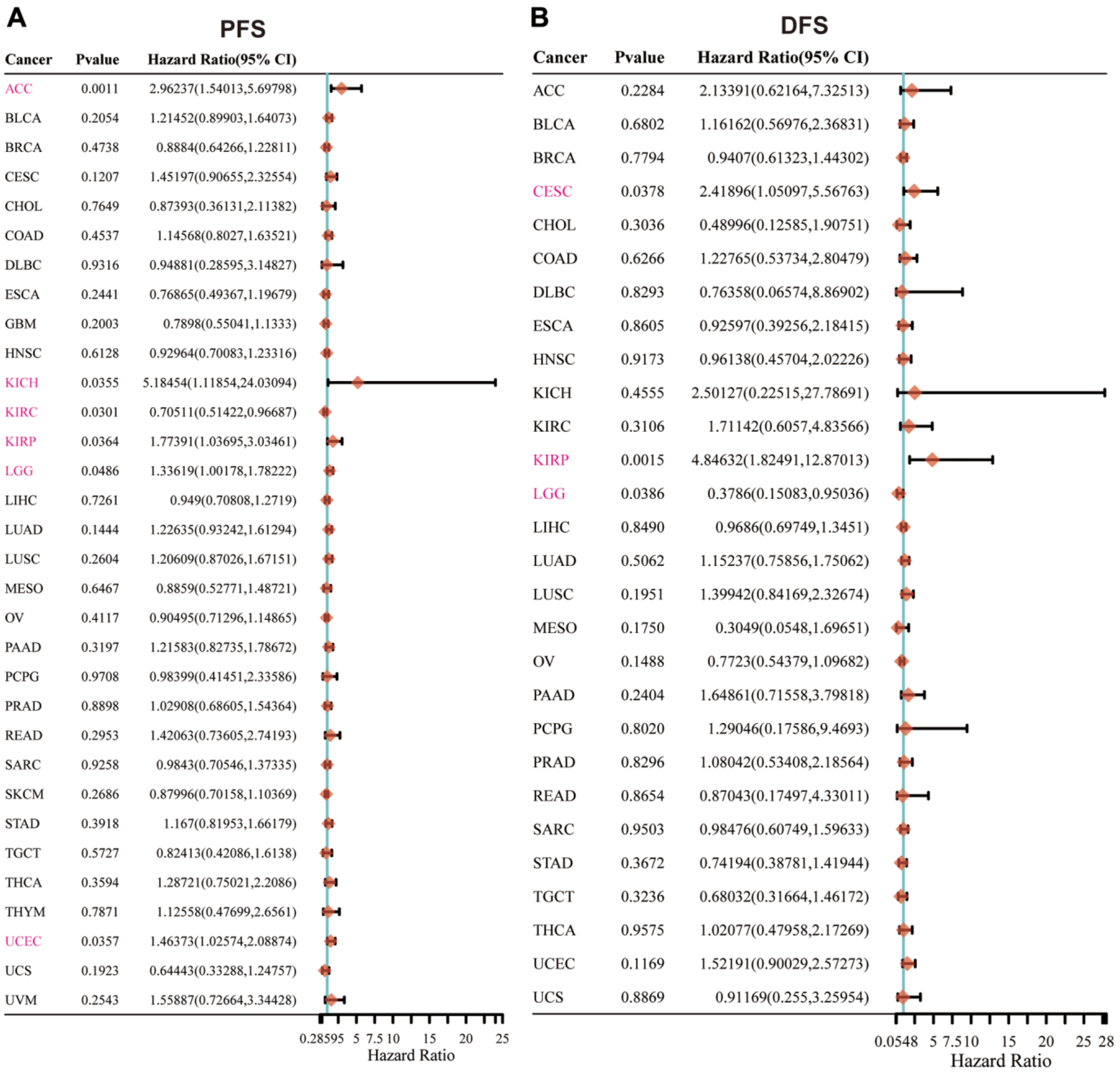

Supplementary Figure 4. Cox risk regression of ADAR in pan-cancer. (**A**, **B**) High ADAR expression predicted poor DFS in CESC (HR = 2.41896, *P* = 0.0378) and KIRP (HR = 4.84632, *P* = 0.0015), but was positively associated with better DFS in LGG (HR = 0.3786, *P* = 0.0386)
