# Supplementary material for: Clinical significance, tumor immune landscape and immunotherapy responses of ADAR in pan-cancer and its association with proliferation and metastasis of bladder cancer
Source: Aging (Albany NY). 2023 Jul 6;15(13):6302–30. doi: 10.18632/aging.204853 (PMC10373965; doi:10.18632/aging.204853)
Supplement: Supplementary Tables 1-2 [file aging-15-204853-s002.pdf]

## SUPPLEMENTARY TABLES

**Supplementary Table 1. List of the top 100 ADAR-related genes from GEPIA2.0.**

| Gene symbol | Gene ID | PCC |
|---|---|---|
| ISG20L2 | ENSG00000143319.16 | 0.75 |
| YY1AP1 | ENSG00000163374.19 | 0.74 |
| SMG7 | ENSG00000116698.20 | 0.72 |
| EIF2AK2 | ENSG00000055332.16 | 0.7 |
| UBAP2L | ENSG00000143569.18 | 0.7 |
| ATF6 | ENSG00000118217.5 | 0.7 |
| PIP5K1A | ENSG00000143398.19 | 0.7 |
| CEP350 | ENSG00000135837.15 | 0.69 |
| UHMK1 | ENSG00000152332.15 | 0.68 |
| RAB3GAP2 | ENSG00000118873.15 | 0.68 |
| TAF5L | ENSG00000135801.9 | 0.67 |
| COPA | ENSG00000122218.14 | 0.67 |
| RPRD2 | ENSG00000163125.15 | 0.67 |
| GATAD2B | ENSG00000143614.7 | 0.67 |
| RBM12 | ENSG00000244462.7 | 0.67 |
| WDR26 | ENSG00000162923.14 | 0.67 |
| ADNP | ENSG00000101126.15 | 0.67 |
| TROVE2 | ENSG00000116747.12 | 0.67 |
| EXOC8 | ENSG00000116903.7 | 0.67 |
| HNRNPU | ENSG00000153187.16 | 0.66 |
| AHCTF1 | ENSG00000153207.14 | 0.66 |
| UBE2Q1 | ENSG00000160714.9 | 0.66 |
| DIEXF | ENSG00000117597.17 | 0.66 |
| ARNT | ENSG00000143437.20 | 0.66 |
| SPRTN | ENSG00000010072.15 | 0.66 |
| RBM12B | ENSG00000183808.11 | 0.65 |
| ACBD3 | ENSG00000182827.8 | 0.65 |
| ARID4B | ENSG00000054267.20 | 0.65 |
| RBBP5 | ENSG00000117222.13 | 0.65 |
| TRIP12 | ENSG00000153827.13 | 0.65 |
| PRRC2C | ENSG00000117523.15 | 0.65 |
| BLZF1 | ENSG00000117475.13 | 0.64 |
| ASH1L | ENSG00000116539.10 | 0.64 |
| SDE2 | ENSG00000143751.9 | 0.64 |
| POLR3C | ENSG00000186141.8 | 0.64 |
| SP3 | ENSG00000172845.13 | 0.64 |
| TPM3 | ENSG00000143549.19 | 0.64 |
| URB2 | ENSG00000135763.9 | 0.64 |
| SCYL3 | ENSG00000000457.13 | 0.64 |
| HEATR1 | ENSG00000119285.10 | 0.64 |
| GOSR1 | ENSG00000108587.14 | 0.63 |
| POGK | ENSG00000143157.11 | 0.63 |
| ETV3 | ENSG00000117036.11 | 0.63 |
| DTX3L | ENSG00000163840.9 | 0.63 |
| USP37 | ENSG00000135913.10 | 0.63 |
| DEDD | ENSG00000158796.16 | 0.63 |
| BROX | ENSG00000162819.11 | 0.63 |
| BCLAF1 | ENSG00000029363.15 | 0.63 |
| THRAP3 | ENSG00000054118.13 | 0.63 |
| TRMT1L | ENSG00000121486.11 | 0.63 |
| ANGEL2 | ENSG00000174606.12 | 0.63 |
| DDX46 | ENSG00000145833.15 | 0.62 |

| | | |
|---|---|---|
| ZNFX1 | ENSG00000124201.14 | 0.62 |
| UHRF1BP1 | ENSG00000065060.16 | 0.62 |
| NSD1 | ENSG00000165671.18 | 0.62 |
| PRUNE | ENSG00000143363.15 | 0.62 |
| TSNAX | ENSG00000116918.13 | 0.62 |
| SNX27 | ENSG00000143376.12 | 0.62 |
| ZNF669 | ENSG00000188295.14 | 0.62 |
| ZMYM4 | ENSG00000146463.11 | 0.62 |
| ZNF281 | ENSG00000162702.7 | 0.62 |
| OTUD7B | ENSG00000264522.5 | 0.62 |
| ZNF623 | ENSG00000183309.11 | 0.62 |
| NUP153 | ENSG00000124789.11 | 0.62 |
| FAM20B | ENSG00000116199.11 | 0.62 |
| HNRNPK | ENSG00000165119.18 | 0.61 |
| UBN1 | ENSG00000118900.14 | 0.61 |
| GTF3C4 | ENSG00000125484.11 | 0.61 |
| CNOT6 | ENSG00000113300.11 | 0.61 |
| RBM27 | ENSG00000091009.7 | 0.61 |
| ZSCAN29 | ENSG00000140265.12 | 0.61 |
| METTL14 | ENSG00000145388.14 | 0.61 |
| SENP1 | ENSG00000079387.13 | 0.61 |
| CELF1 | ENSG00000149187.17 | 0.61 |
| RFX5 | ENSG00000143390.17 | 0.61 |
| IARS2 | ENSG00000067704.9 | 0.61 |
| GPR89A | ENSG00000117262.18 | 0.61 |
| CREB1 | ENSG00000118260.14 | 0.61 |
| WAC | ENSG00000095787.21 | 0.61 |
| CACUL1 | ENSG00000151893.14 | 0.61 |
| SLC25A44 | ENSG00000160785.13 | 0.6 |
| PUM2 | ENSG00000055917.15 | 0.6 |
| CDC73 | ENSG00000134371.9 | 0.6 |
| POGZ | ENSG00000143442.21 | 0.6 |
| ZBTB41 | ENSG00000177888.7 | 0.6 |
| PYGO2 | ENSG00000163348.3 | 0.6 |
| NUP133 | ENSG00000069248.11 | 0.6 |
| UBQLN4 | ENSG00000160803.7 | 0.6 |
| STX17 | ENSG00000136874.10 | 0.6 |
| YME1L1 | ENSG00000136758.18 | 0.6 |
| CSNK1G1 | ENSG00000169118.15 | 0.6 |
| 7-Mar | ENSG00000136536.14 | 0.6 |
| SIN3A | ENSG00000169375.15 | 0.6 |
| KDM5B | ENSG00000117139.16 | 0.6 |
| CDC42SE1 | ENSG00000197622.12 | 0.6 |
| CAPRIN1 | ENSG00000135387.19 | 0.6 |
| INTS7 | ENSG00000143493.12 | 0.59 |
| SMEK2 | ENSG00000275052.4 | 0.59 |
| FBXW2 | ENSG00000119402.16 | 0.59 |
| PGGT1B | ENSG00000164219.9 | 0.59 |

**Supplementary Table 2. Correlation analysis between ADAR expression and immune cell clustering.**

| ID | Main specificity | Main function | #Genes | Annotation reliability |
|---|---|---|---|---|
| 1 | T-cells | Immune response | 378 | High |
| 2 | T-cells | T-cell receptor | 243 | Medium |
| 3 | Suprabasal keratinocytes | Cornification | 185 | High |
| 4 | Platelets | Platelet activation | 157 | High |
| 5 | Bronchus | Unknown function | 208 | Low |
| 6 | Late spermatids | Spermatogenesis | 433 | Medium |
| 7 | NK-cells | Immune response regulation | 312 | Medium |
| 8 | Oligodendrocytes | Myelin sheath organization | 301 | Medium |
| 9 | Neurons | Nervous system development | 235 | Medium |
| 10 | Neurons and Oligodendrocytes | Nervous system development | 262 | High |
| 11 | Non-specific | Transcription | 188 | Medium |
| 12 | Non-specific | Chromatin organization | 249 | Low |
| 13 | Hepatocytes | Oxidoreductase activity | 177 | High |
| 14 | Endometrium | Transcription regulation | 304 | Low |
| 15 | Smooth muscle cells | Muscle contraction | 246 | High |
| 16 | Non-specific | RNA binding | 751 | Low |
| 17 | Granulosa cells | Transcription regulation | 353 | Medium |
| 18 | Non-specific | Mixed function | 294 | Low |
| 19 | Macrophages | Innate immune response | 247 | High |
| 20 | Airway and Pancreas | Proteolysis | 48 | Medium |
| 21 | Serous glandular cells | Salivary secretion | 134 | Medium |
| 22 | Squamous epithelial cells | Keratinization | 298 | High |
| 23 | Langerhans cells | Immune response | 91 | Medium |
| 24 | Spermatids | Unknown function | 117 | Low |
| 25 | Schwann cells and Melanocytes | Mixed function | 80 | Low |
| 26 | Intestinal epithelial cells | Absorption | 271 | High |
| 27 | Skeletal myocytes | Muscle contraction | 105 | High |
| 28 | Cytotrophoblasts | Unknown function | 227 | Low |
| 29 | Hepatocytes | Metabolism | 152 | Medium |
| 30 | Breast | Lactation | 82 | High |
| 31 | Spermatocytes | Spermatogenesis | 213 | High |
| 32 | Neurons | Synaptic function | 237 | High |
| 33 | Macrophages | Degranulation | 283 | High |
| 34 | Plasma cells | Humoral immune response | 86 | High |
| 35 | Bipolar cells | Visual perception | 159 | High |
| 36 | Spermatids | Spermatogenesis | 419 | High |
| 37 | Neurons | Neuronal signaling | 496 | High |
| 38 | Neurons | Neuronal signaling | 626 | High |
| 39 | Non-specific | Translation | 186 | Medium |
| 40 | Plasma cells | Humoral immune response | 409 | Medium |
| 41 | Alveolar cells | Lung function | 118 | Medium |
| 42 | Spermatocytes and Spermatids | Spermatogenesis | 353 | Medium |

| | | | | |
|---|---|---|---|---|
| 43 | Non-specific | Cell proliferation | 263 | Medium |
| 44 | Photoreceptor cells | Visual perception | 359 | High |
| 45 | Plasma cells | Humoral immune response | 78 | High |
| 46 | Granulocytes | Mast cell degranulation | 60 | Medium |
| 47 | Spermatogonia | Spermatogenesis | 119 | Medium |
| 48 | Plasmacytoid dendritic cells | Unknown function | 134 | Low |
| 49 | Early spermatids | Spermatogenesis | 272 | High |
| 50 | Enterocytes | Absorption | 308 | Medium |
| 51 | Late spermatids | Spermatogenesis | 389 | High |
| 52 | Endometrial stromal cells | ECM organization | 116 | Medium |
| 53 | Connective tissue cells | ECM organization | 187 | Medium |
| 54 | Extravillous trophoblasts | Unknown function | 188 | Medium |
| 55 | Endocrine cells | Hormone signaling | 81 | High |
| 56 | Ciliated cells | Cilium assembly | 425 | High |
| 57 | Early spermatids | Flagellum and Golgi organization | 325 | Medium |
| 58 | Gastric mucus-secreting cells | Digestion | 45 | High |
| 59 | Proximal enterocytes | Transmembrane transport | 209 | High |
| 60 | Proximal tubular cells | Absorption | 361 | High |
| 61 | Non-specific | Transcription | 366 | Low |
| 62 | B-cells | B-cell activation | 166 | High |
| 63 | Astrocytes | Nervous system maintenance | 208 | High |
| 64 | Non-specific | Transcription | 655 | Medium |
| 65 | Fibroblasts | ECM organization | 292 | High |
| 66 | Endothelial cells | Angiogenesis | 468 | Medium |
| 67 | Syncytiotrophoblasts | Pregnancy | 153 | Medium |
| 68 | Muller glia cells | Visual perception | 142 | High |
| 69 | Macrophages | Innate immune response | 217 | High |
| 70 | Basal prostatic cells | Lipid metabolism | 167 | Medium |
| 71 | Erythroid cells | Oxygen transport | 140 | High |
| 72 | Smooth muscle cells | ECM organization | 208 | High |
| 73 | Prostatic glandular cells | Transcription | 109 | High |
| 74 | Pancreatic cells | Mixed function | 331 | Low |
| 75 | Myeloid cells | Innate immune response | 390 | Medium |
| 76 | Spermatocytes and Spermatogonia | Spermatogenesis | 164 | High |
| 77 | Cardiomyocytes | Muscle contraction | 404 | High |
