# Supplementary material for: Clinical significance, tumor immune landscape and immunotherapy responses of ADAR in pan-cancer and its association with proliferation and metastasis of bladder cancer
Source: Aging (Albany NY). 2023 Jul 6;15(13):6302–30. doi: 10.18632/aging.204853 (PMC10373965; doi:10.18632/aging.204853)
Supplement: Supplementary Table 3 [file aging-15-204853-s003.docx]

| **Supplementary Table 3. Differentially expressed genes between the ADAR high-expression group and the ADAR low-expression group.** | | | | | | |
| --- | --- | --- | --- | --- | --- | --- |
|  | logFC | AveExpr | t | P.Value | adj.P.Val | B |
| SPINK1 | -2.68528 | 6.270223 | -6.59616 | 1.32E-10 | 5.09E-10 | 13.45192 |
| UPK1A | -2.52448 | 5.400612 | -7.33605 | 1.20E-12 | 5.83E-12 | 18.03415 |
| UPK2 | -2.32663 | 7.160026 | -6.55601 | 1.68E-10 | 6.44E-10 | 13.21435 |
| PSCA | -2.24013 | 7.966085 | -6.21596 | 1.27E-09 | 4.46E-09 | 11.24969 |
| DHRS2 | -2.20026 | 6.058669 | -6.60715 | 1.23E-10 | 4.78E-10 | 13.51714 |
| UPK3A | -1.94674 | 4.792121 | -6.06464 | 3.03E-09 | 1.03E-08 | 10.40317 |
| VSIG2 | -1.88895 | 6.294573 | -6.82693 | 3.17E-11 | 1.32E-10 | 14.83991 |
| HMGCS2 | -1.86144 | 4.719247 | -5.2617 | 2.31E-07 | 6.55E-07 | 6.2081 |
| BTBD16 | -1.68685 | 3.476676 | -6.39181 | 4.50E-10 | 1.66E-09 | 12.25499 |
| GPX2 | -1.66047 | 7.176261 | -5.10925 | 4.98E-07 | 1.37E-06 | 5.469775 |
| S100P | -1.5816 | 10.71472 | -5.85678 | 9.75E-09 | 3.18E-08 | 9.268767 |
| SNX31 | -1.55968 | 4.000958 | -5.57131 | 4.61E-08 | 1.41E-07 | 7.765435 |
| SLC14A1 | -1.51442 | 3.585346 | -6.0612 | 3.09E-09 | 1.05E-08 | 10.38408 |
| SNCG | -1.4978 | 8.47955 | -6.37862 | 4.87E-10 | 1.79E-09 | 12.17879 |
| KRT20 | -1.45941 | 4.893459 | -3.87117 | 0.000126 | 0.00026 | 0.199677 |
| TESC | -1.38651 | 3.521669 | -6.63617 | 1.03E-10 | 4.04E-10 | 13.68981 |
| FXYD4 | -1.35188 | 2.72263 | -5.38326 | 1.24E-07 | 3.61E-07 | 6.810374 |
| PLA2G2F | -1.32168 | 3.867016 | -5.26259 | 2.30E-07 | 6.52E-07 | 6.212481 |
| FAM3B | -1.30798 | 3.965073 | -5.38876 | 1.20E-07 | 3.51E-07 | 6.837886 |
| CYP4B1 | -1.27843 | 5.398347 | -4.34574 | 1.76E-05 | 4.05E-05 | 2.063769 |
| AKR1C2 | -1.27772 | 4.891557 | -4.98326 | 9.27E-07 | 2.47E-06 | 4.874032 |
| C10orf99 | -1.27625 | 5.286498 | -3.97257 | 8.41E-05 | 0.000178 | 0.581285 |
| UPK1B | -1.27452 | 6.197454 | -3.65367 | 0.000292 | 0.000574 | -0.58786 |
| TAC3 | -1.2374 | 1.992034 | -4.48474 | 9.51E-06 | 2.26E-05 | 2.646999 |
| HSD17B2 | -1.23367 | 2.523804 | -6.96888 | 1.30E-11 | 5.63E-11 | 15.71241 |
| TBX2 | -1.21311 | 4.844865 | -6.43293 | 3.52E-10 | 1.32E-09 | 12.49336 |
| MAL | -1.21106 | 3.956706 | -4.59639 | 5.75E-06 | 1.40E-05 | 3.127448 |
| HS3ST6 | -1.21015 | 2.283694 | -6.13196 | 2.06E-09 | 7.11E-09 | 10.77764 |
| PM20D1 | -1.20895 | 2.980617 | -3.94165 | 9.53E-05 | 0.0002 | 0.463953 |
| BCAS1 | -1.19048 | 3.578225 | -5.33964 | 1.55E-07 | 4.48E-07 | 6.592867 |
| SCNN1B | -1.18203 | 4.54381 | -4.90638 | 1.34E-06 | 3.52E-06 | 4.51695 |
| GDF15 | -1.14967 | 6.749212 | -5.02916 | 7.40E-07 | 1.99E-06 | 5.089557 |
| FABP4 | -1.14847 | 4.209602 | -3.78907 | 0.000174 | 0.000352 | -0.10257 |
| TNNC1 | -1.11659 | 3.308508 | -5.64723 | 3.07E-08 | 9.52E-08 | 8.158977 |
| SMIM22 | -1.11629 | 4.226409 | -6.87066 | 2.41E-11 | 1.02E-10 | 15.10719 |
| REEP6 | -1.1111 | 5.327865 | -6.49999 | 2.36E-10 | 8.91E-10 | 12.88484 |
| TNNT3 | -1.10472 | 3.021801 | -4.36916 | 1.59E-05 | 3.67E-05 | 2.160886 |
| CAPS | -1.10418 | 6.086618 | -5.67319 | 2.67E-08 | 8.33E-08 | 8.294623 |
| PADI3 | -1.08277 | 4.971769 | -3.52778 | 0.000467 | 0.000892 | -1.02425 |
| TMPRSS2 | -1.0821 | 4.05988 | -4.59072 | 5.90E-06 | 1.43E-05 | 3.102816 |
| CYP4F22 | -1.07173 | 3.613065 | -4.32737 | 1.90E-05 | 4.37E-05 | 1.987976 |
| PVALB | -1.07082 | 1.880575 | -4.73049 | 3.09E-06 | 7.78E-06 | 3.718581 |
| TBX1 | -1.05677 | 3.423554 | -5.33866 | 1.56E-07 | 4.50E-07 | 6.588038 |
| BHMT | -1.0466 | 2.426039 | -4.00957 | 7.24E-05 | 0.000154 | 0.722798 |
| HPGD | -1.04148 | 5.172833 | -3.77291 | 0.000185 | 0.000373 | -0.16135 |
| ANXA10 | -1.03397 | 2.682579 | -3.48037 | 0.000555 | 0.00105 | -1.18484 |
| CRTAC1 | -1.0279 | 2.154303 | -4.22962 | 2.89E-05 | 6.49E-05 | 1.589429 |
| FABP6 | -1.02677 | 3.959825 | -4.92564 | 1.23E-06 | 3.22E-06 | 4.605927 |
| BAMBI | -1.01521 | 5.269421 | -4.73769 | 2.99E-06 | 7.53E-06 | 3.75078 |
| SCNN1G | -1.00502 | 3.669745 | -4.42119 | 1.26E-05 | 2.95E-05 | 2.378281 |
| KIF23 | 1.000377 | 3.876113 | 11.08571 | 3.92E-25 | 1.13E-23 | 46.30501 |
| CKAP2L | 1.001959 | 3.156683 | 10.79407 | 4.71E-24 | 1.14E-22 | 43.85412 |
| CD163 | 1.003007 | 3.224107 | 5.392311 | 1.18E-07 | 3.45E-07 | 6.855689 |
| NMU | 1.003655 | 3.23301 | 5.411683 | 1.07E-07 | 3.14E-07 | 6.952911 |
| LAG3 | 1.003784 | 2.453768 | 6.7998 | 3.75E-11 | 1.55E-10 | 14.67474 |
| AHNAK2 | 1.004122 | 2.443568 | 5.444091 | 9.02E-08 | 2.67E-07 | 7.116229 |
| ADCY7 | 1.004152 | 1.62164 | 10.70145 | 1.03E-23 | 2.34E-22 | 43.08322 |
| FN1 | 1.006688 | 7.168709 | 4.322306 | 1.94E-05 | 4.46E-05 | 1.967113 |
| CERS2 | 1.008387 | 5.316335 | 11.3512 | 3.97E-26 | 1.41E-24 | 48.56616 |
| BUB1B | 1.01123 | 3.561076 | 10.61148 | 2.19E-23 | 4.70E-22 | 42.338 |
| STAG1 | 1.012099 | 3.311888 | 13.45118 | 2.24E-34 | 8.10E-32 | 67.32058 |
| NCAPG | 1.013165 | 3.572695 | 10.35206 | 1.90E-22 | 3.42E-21 | 40.20917 |
| C12orf75 | 1.013484 | 3.805145 | 6.456963 | 3.05E-10 | 1.14E-09 | 12.63328 |
| IQGAP3 | 1.014148 | 4.191072 | 10.41615 | 1.12E-22 | 2.10E-21 | 40.73231 |
| BNC1 | 1.016159 | 1.298168 | 5.141112 | 4.25E-07 | 1.17E-06 | 5.622525 |
| DCBLD2 | 1.016872 | 3.374909 | 7.618254 | 1.82E-13 | 9.59E-13 | 19.88037 |
| CDCA8 | 1.017964 | 5.230266 | 10.60268 | 2.36E-23 | 5.00E-22 | 42.26533 |
| B2M | 1.018194 | 10.09854 | 9.060802 | 5.51E-18 | 4.97E-17 | 30.09379 |
| PMAIP1 | 1.018635 | 4.246931 | 7.795373 | 5.44E-14 | 3.03E-13 | 21.06572 |
| INCENP | 1.019677 | 4.41324 | 12.71302 | 2.08E-31 | 3.05E-29 | 60.56943 |
| MOB3B | 1.020142 | 2.531993 | 9.431756 | 3.14E-19 | 3.39E-18 | 32.91305 |
| MSH6 | 1.020238 | 4.123379 | 13.58622 | 6.31E-35 | 2.98E-32 | 68.5716 |
| EIF2AK2 | 1.021548 | 4.524208 | 16.49462 | 3.64E-47 | 3.10E-43 | 96.42291 |
| GPRIN1 | 1.022408 | 2.548015 | 9.16726 | 2.44E-18 | 2.31E-17 | 30.89532 |
| BTN3A3 | 1.022458 | 3.577096 | 11.05721 | 5.01E-25 | 1.42E-23 | 46.06396 |
| CDC25B | 1.023698 | 5.644345 | 8.725298 | 6.90E-17 | 5.38E-16 | 27.60874 |
| NCAPG2 | 1.023738 | 3.891802 | 12.00697 | 1.23E-28 | 7.99E-27 | 54.26627 |
| CXCL8 | 1.026029 | 4.60799 | 4.752947 | 2.79E-06 | 7.04E-06 | 3.819081 |
| NUP153 | 1.026693 | 4.897878 | 13.91099 | 2.95E-36 | 2.28E-33 | 71.5991 |
| ROBO1 | 1.02732 | 3.274876 | 7.426345 | 6.62E-13 | 3.29E-12 | 18.61913 |
| SPRR2A | 1.028457 | 2.353133 | 3.677211 | 0.000267 | 0.000527 | -0.50469 |
| KIF4A | 1.028835 | 4.271094 | 10.45205 | 8.29E-23 | 1.58E-21 | 41.02616 |
| PLK1 | 1.029301 | 4.569417 | 10.03385 | 2.58E-21 | 3.82E-20 | 37.63999 |
| RNF213 | 1.029589 | 4.725543 | 12.06838 | 7.11E-29 | 5.02E-27 | 54.80789 |
| NRIP1 | 1.033093 | 3.925511 | 9.405639 | 3.85E-19 | 4.10E-18 | 32.71216 |
| NCAPD2 | 1.033883 | 5.834284 | 12.81494 | 8.17E-32 | 1.36E-29 | 61.49224 |
| HLA-F | 1.034834 | 5.208958 | 6.755436 | 4.94E-11 | 2.02E-10 | 14.40581 |
| PDIA6 | 1.0351 | 6.825739 | 11.22477 | 1.19E-25 | 3.75E-24 | 47.48585 |
| KLF11 | 1.035206 | 3.429744 | 11.69773 | 1.91E-27 | 9.08E-26 | 51.55857 |
| C1QC | 1.035481 | 6.868777 | 5.173621 | 3.61E-07 | 1.00E-06 | 5.77922 |
| SLC7A1 | 1.03582 | 4.622037 | 9.496024 | 1.89E-19 | 2.11E-18 | 33.40887 |
| A2ML1 | 1.037471 | 3.073575 | 4.636216 | 4.79E-06 | 1.18E-05 | 3.301433 |
| CCL4 | 1.040043 | 2.889762 | 6.610765 | 1.20E-10 | 4.68E-10 | 13.53861 |
| MAFB | 1.040192 | 4.600343 | 7.561066 | 2.68E-13 | 1.39E-12 | 19.50199 |
| CEBPZ | 1.041064 | 4.452317 | 12.83865 | 6.57E-32 | 1.13E-29 | 61.70741 |
| IL1RAP | 1.04178 | 2.520557 | 9.155138 | 2.68E-18 | 2.52E-17 | 30.80374 |
| SLFN13 | 1.044914 | 2.5151 | 7.517997 | 3.59E-13 | 1.83E-12 | 19.21845 |
| ITGB8 | 1.045565 | 2.459493 | 8.396806 | 7.72E-16 | 5.37E-15 | 25.23797 |
| H2BC12 | 1.045792 | 6.796392 | 7.574465 | 2.45E-13 | 1.27E-12 | 19.59046 |
| CHST11 | 1.046011 | 3.60061 | 7.019648 | 9.38E-12 | 4.14E-11 | 16.02793 |
| FGD6 | 1.046634 | 2.751912 | 12.62816 | 4.52E-31 | 5.91E-29 | 59.80347 |
| DPYD | 1.048376 | 2.749712 | 7.435017 | 6.25E-13 | 3.11E-12 | 18.6756 |
| SPIN4 | 1.048862 | 3.017963 | 9.979405 | 4.01E-21 | 5.73E-20 | 37.20517 |
| CDCA7 | 1.049014 | 3.873072 | 8.845582 | 2.81E-17 | 2.32E-16 | 28.49241 |
| NOTCH2 | 1.049128 | 3.770044 | 12.53953 | 1.01E-30 | 1.20E-28 | 59.00579 |
| STIL | 1.049362 | 2.903875 | 13.72895 | 1.65E-35 | 1.00E-32 | 69.89896 |
| CIP2A | 1.049366 | 3.280478 | 12.78889 | 1.04E-31 | 1.70E-29 | 61.25608 |
| CD8A | 1.049557 | 2.620728 | 6.951007 | 1.45E-11 | 6.27E-11 | 15.6018 |
| DSG2 | 1.050462 | 6.161597 | 8.621955 | 1.49E-16 | 1.11E-15 | 26.85613 |
| KIF14 | 1.051839 | 2.60909 | 12.23601 | 1.58E-29 | 1.36E-27 | 56.29308 |
| MICB | 1.052229 | 3.071288 | 8.744849 | 5.97E-17 | 4.69E-16 | 27.75181 |
| HLA-B | 1.052804 | 10.34581 | 7.605581 | 1.99E-13 | 1.04E-12 | 19.79634 |
| USP1 | 1.052962 | 5.238174 | 12.45653 | 2.15E-30 | 2.32E-28 | 58.26108 |
| HLA-DRB1 | 1.053777 | 8.791652 | 5.539749 | 5.45E-08 | 1.65E-07 | 7.603184 |
| SLFN11 | 1.053896 | 3.024151 | 7.898439 | 2.66E-14 | 1.54E-13 | 21.76473 |
| GZMB | 1.055454 | 3.28354 | 5.399062 | 1.14E-07 | 3.34E-07 | 6.889536 |
| SMC2 | 1.056628 | 3.920307 | 12.32762 | 6.91E-30 | 6.61E-28 | 57.10867 |
| IL2RB | 1.057938 | 2.74958 | 8.104223 | 6.29E-15 | 3.89E-14 | 23.18039 |
| RAD51AP1 | 1.058436 | 3.905728 | 10.71482 | 9.20E-24 | 2.12E-22 | 43.19431 |
| CYBB | 1.058577 | 3.583617 | 6.304561 | 7.54E-10 | 2.72E-09 | 11.75332 |
| SPRR1B | 1.058711 | 4.582522 | 3.199661 | 0.001484 | 0.002661 | -2.09368 |
| MYO1B | 1.061944 | 4.424166 | 8.766063 | 5.10E-17 | 4.03E-16 | 27.90731 |
| CGAS | 1.063875 | 3.230343 | 10.11987 | 1.28E-21 | 1.98E-20 | 38.32977 |
| SERPINB9 | 1.064825 | 3.725244 | 7.809228 | 4.94E-14 | 2.77E-13 | 21.15929 |
| FAT2 | 1.065585 | 2.847442 | 5.463627 | 8.15E-08 | 2.42E-07 | 7.215081 |
| GSDMC | 1.06611 | 2.56755 | 5.649912 | 3.02E-08 | 9.39E-08 | 8.172987 |
| HLA-DQA1 | 1.066729 | 4.271735 | 5.719529 | 2.07E-08 | 6.54E-08 | 8.538005 |
| ANXA3 | 1.073487 | 3.480721 | 6.156755 | 1.78E-09 | 6.19E-09 | 10.9164 |
| FAM83D | 1.075032 | 5.204597 | 9.458987 | 2.53E-19 | 2.77E-18 | 33.12286 |
| APOL6 | 1.07643 | 4.593499 | 11.14431 | 2.37E-25 | 7.12E-24 | 46.80162 |
| LAP3 | 1.077989 | 6.056429 | 11.16921 | 1.91E-25 | 5.84E-24 | 47.01311 |
| M6PR | 1.079613 | 5.598331 | 12.42904 | 2.76E-30 | 2.94E-28 | 58.01485 |
| SERPINB13 | 1.083233 | 1.969099 | 4.792508 | 2.31E-06 | 5.90E-06 | 3.997124 |
| BST2 | 1.083843 | 8.35196 | 5.809849 | 1.26E-08 | 4.08E-08 | 9.017234 |
| CCNA2 | 1.08451 | 4.921916 | 10.71998 | 8.80E-24 | 2.04E-22 | 43.2372 |
| MKI67 | 1.084797 | 4.760765 | 10.93128 | 1.47E-24 | 3.87E-23 | 45.00277 |
| OXCT1 | 1.085991 | 3.681558 | 7.23339 | 2.36E-12 | 1.11E-11 | 17.37574 |
| DLGAP5 | 1.086301 | 4.314032 | 10.42792 | 1.01E-22 | 1.91E-21 | 40.8286 |
| NUAK2 | 1.086977 | 2.951262 | 8.915574 | 1.66E-17 | 1.41E-16 | 29.01037 |
| CENPF | 1.088918 | 4.35458 | 10.98951 | 8.94E-25 | 2.42E-23 | 45.4926 |
| PKP1 | 1.093526 | 4.966882 | 4.652483 | 4.44E-06 | 1.10E-05 | 3.372874 |
| PRAME | 1.093778 | 1.522843 | 5.207894 | 3.04E-07 | 8.50E-07 | 5.945358 |
| TXNRD1 | 1.097067 | 4.972354 | 8.948397 | 1.29E-17 | 1.12E-16 | 29.25421 |
| RACGAP1 | 1.100806 | 4.977649 | 12.2527 | 1.36E-29 | 1.19E-27 | 56.44153 |
| TNFAIP3 | 1.101011 | 4.70217 | 8.345263 | 1.12E-15 | 7.65E-15 | 24.87174 |
| HLA-DRB5 | 1.101151 | 6.899984 | 5.45735 | 8.42E-08 | 2.50E-07 | 7.183288 |
| CNOT1 | 1.10133 | 4.811105 | 11.97343 | 1.66E-28 | 1.03E-26 | 53.9709 |
| E2F7 | 1.102456 | 2.381287 | 11.38195 | 3.04E-26 | 1.13E-24 | 48.82983 |
| FKBP5 | 1.104464 | 3.494774 | 8.417001 | 6.67E-16 | 4.67E-15 | 25.38189 |
| MSN | 1.112388 | 6.513957 | 7.657234 | 1.40E-13 | 7.46E-13 | 20.1395 |
| BIRC3 | 1.113571 | 3.157765 | 7.620902 | 1.79E-13 | 9.43E-13 | 19.89794 |
| CDCA7L | 1.116086 | 2.256854 | 7.659904 | 1.37E-13 | 7.33E-13 | 20.15728 |
| DTX3L | 1.116466 | 5.555216 | 14.98385 | 1.00E-40 | 2.84E-37 | 81.76636 |
| SAMHD1 | 1.117518 | 4.339514 | 10.69351 | 1.10E-23 | 2.48E-22 | 43.01735 |
| FAM111B | 1.118092 | 3.545194 | 10.204 | 6.43E-22 | 1.06E-20 | 39.0079 |
| PPP1R14C | 1.119594 | 3.10633 | 5.318047 | 1.73E-07 | 4.98E-07 | 6.48579 |
| MMP12 | 1.124238 | 4.023405 | 4.670093 | 4.10E-06 | 1.02E-05 | 3.450471 |
| KRT6C | 1.125831 | 1.592554 | 4.538305 | 7.48E-06 | 1.80E-05 | 2.876175 |
| ITGA2 | 1.12901 | 4.325406 | 7.925801 | 2.20E-14 | 1.28E-13 | 21.95144 |
| HLA-DPA1 | 1.132437 | 5.654515 | 6.129885 | 2.08E-09 | 7.19E-09 | 10.76602 |
| TAP2 | 1.135932 | 4.219616 | 11.29577 | 6.41E-26 | 2.19E-24 | 48.09177 |
| ASPM | 1.137673 | 3.082109 | 12.08956 | 5.88E-29 | 4.26E-27 | 54.99505 |
| NLRC5 | 1.138375 | 3.191261 | 10.38594 | 1.44E-22 | 2.65E-21 | 40.48546 |
| MELK | 1.144209 | 4.665148 | 10.28914 | 3.19E-22 | 5.53E-21 | 39.69747 |
| TPX2 | 1.14434 | 6.345757 | 10.13065 | 1.17E-21 | 1.83E-20 | 38.41651 |
| CCL20 | 1.150932 | 2.638592 | 5.586566 | 4.25E-08 | 1.30E-07 | 7.844166 |
| TMEM45A | 1.154895 | 3.739229 | 5.783685 | 1.46E-08 | 4.68E-08 | 8.87776 |
| EGFR | 1.155624 | 4.753011 | 7.747058 | 7.58E-14 | 4.16E-13 | 20.74037 |
| CDCP1 | 1.158551 | 5.153891 | 10.25855 | 4.11E-22 | 6.95E-21 | 39.44927 |
| ASAP2 | 1.158697 | 2.864414 | 12.10383 | 5.18E-29 | 3.80E-27 | 55.12123 |
| PRRC2B | 1.162188 | 4.331019 | 13.52069 | 1.17E-34 | 4.84E-32 | 67.96391 |
| B3GNT5 | 1.164015 | 3.2352 | 11.51091 | 9.86E-27 | 4.10E-25 | 49.93972 |
| FGFBP1 | 1.165042 | 3.675884 | 3.920346 | 0.000104 | 0.000217 | 0.38361 |
| XAF1 | 1.165326 | 2.196068 | 10.50665 | 5.27E-23 | 1.05E-21 | 41.47412 |
| HELZ2 | 1.167459 | 4.109814 | 13.96086 | 1.84E-36 | 1.56E-33 | 72.06623 |
| HLTF | 1.167624 | 3.073086 | 11.39175 | 2.79E-26 | 1.05E-24 | 48.91396 |
| FYB1 | 1.169787 | 2.418014 | 8.06256 | 8.44E-15 | 5.13E-14 | 22.89164 |
| F3 | 1.172781 | 4.959372 | 5.870172 | 9.05E-09 | 2.96E-08 | 9.340836 |
| TRIM22 | 1.174599 | 4.232838 | 8.522085 | 3.10E-16 | 2.24E-15 | 26.13469 |
| CEACAM5 | 1.174601 | 3.00871 | 4.149969 | 4.05E-05 | 8.92E-05 | 1.270821 |
| SEC24C | 1.17742 | 4.204877 | 12.39114 | 3.89E-30 | 3.94E-28 | 57.67588 |
| TOP2A | 1.179639 | 6.164625 | 10.77191 | 5.68E-24 | 1.36E-22 | 43.66935 |
| PLAAT4 | 1.180105 | 5.901897 | 5.846317 | 1.03E-08 | 3.35E-08 | 9.212529 |
| CDKN2A | 1.185102 | 3.584904 | 4.550625 | 7.07E-06 | 1.70E-05 | 2.929233 |
| TNC | 1.186212 | 4.261984 | 5.700638 | 2.30E-08 | 7.23E-08 | 8.438582 |
| LCP1 | 1.186962 | 5.782066 | 7.646343 | 1.51E-13 | 8.02E-13 | 20.067 |
| DEK | 1.190167 | 5.980873 | 13.0713 | 7.68E-33 | 1.98E-30 | 63.82706 |
| OSMR | 1.190191 | 3.994673 | 7.613065 | 1.89E-13 | 9.91E-13 | 19.84595 |
| ANLN | 1.191521 | 4.751824 | 10.40906 | 1.19E-22 | 2.21E-21 | 40.67432 |
| CTSV | 1.194147 | 2.75687 | 7.23449 | 2.34E-12 | 1.10E-11 | 17.38275 |
| ADAR | 1.1962 | 6.686639 | 24.46987 | 6.02E-82 | 1.02E-77 | 175.4882 |
| ARNTL2 | 1.199907 | 3.970722 | 10.33851 | 2.13E-22 | 3.78E-21 | 40.09881 |
| HLA-DRA | 1.203923 | 9.310794 | 6.311042 | 7.25E-10 | 2.62E-09 | 11.79039 |
| IFITM1 | 1.207449 | 6.535017 | 7.118188 | 4.98E-12 | 2.26E-11 | 16.6454 |
| ECT2 | 1.21144 | 4.594958 | 12.6302 | 4.43E-31 | 5.84E-29 | 59.82184 |
| RECQL | 1.220822 | 3.760648 | 14.11557 | 4.22E-37 | 4.22E-34 | 73.51893 |
| RHCG | 1.22348 | 3.233285 | 4.951339 | 1.08E-06 | 2.86E-06 | 4.725167 |
| KRT14 | 1.22901 | 5.465987 | 3.101094 | 0.002062 | 0.003629 | -2.39558 |
| ZNF770 | 1.229239 | 3.072503 | 11.46226 | 1.51E-26 | 6.02E-25 | 49.52029 |
| DSC3 | 1.229304 | 3.575642 | 4.350762 | 1.72E-05 | 3.96E-05 | 2.084567 |
| KRT16 | 1.230135 | 6.105673 | 3.783432 | 0.000178 | 0.000359 | -0.12312 |
| BATF2 | 1.231704 | 2.936732 | 8.140984 | 4.84E-15 | 3.04E-14 | 23.43604 |
| OR2I1P | 1.236442 | 2.038177 | 7.216382 | 2.64E-12 | 1.23E-11 | 17.26734 |
| IFI16 | 1.241517 | 5.769126 | 8.076189 | 7.66E-15 | 4.69E-14 | 22.98598 |
| ITPR3 | 1.249253 | 4.583669 | 12.33147 | 6.68E-30 | 6.45E-28 | 57.14307 |
| CD109 | 1.254964 | 2.956612 | 7.584392 | 2.29E-13 | 1.19E-12 | 19.65607 |
| UBE2L6 | 1.256399 | 7.019656 | 10.9236 | 1.57E-24 | 4.12E-23 | 44.93828 |
| HERC5 | 1.266428 | 2.775991 | 10.15721 | 9.43E-22 | 1.50E-20 | 38.63036 |
| PARP14 | 1.268742 | 5.027058 | 16.07699 | 2.27E-45 | 1.29E-41 | 92.33935 |
| TNFSF10 | 1.269618 | 6.416572 | 8.007015 | 1.25E-14 | 7.46E-14 | 22.50837 |
| FCGR3A | 1.274594 | 4.665299 | 6.353921 | 5.63E-10 | 2.06E-09 | 12.03645 |
| IFIT2 | 1.278937 | 3.284512 | 9.076173 | 4.90E-18 | 4.45E-17 | 30.20914 |
| DDX58 | 1.282309 | 4.033734 | 12.09642 | 5.53E-29 | 4.04E-27 | 55.05567 |
| CXCL1 | 1.292254 | 4.43144 | 5.491832 | 7.02E-08 | 2.10E-07 | 7.358342 |
| CDH3 | 1.298694 | 5.412386 | 6.50732 | 2.25E-10 | 8.55E-10 | 12.9278 |
| CXCL13 | 1.302357 | 3.640385 | 5.555282 | 5.02E-08 | 1.53E-07 | 7.682943 |
| CMPK2 | 1.31625 | 3.372138 | 11.5556 | 6.67E-27 | 2.89E-25 | 50.32582 |
| PARP9 | 1.316365 | 4.584006 | 14.50598 | 1.01E-38 | 2.15E-35 | 77.20846 |
| SMC4 | 1.324079 | 4.071345 | 13.8327 | 6.18E-36 | 4.38E-33 | 70.86693 |
| HERC6 | 1.324956 | 3.527751 | 11.33475 | 4.57E-26 | 1.60E-24 | 48.42527 |
| GBP4 | 1.326995 | 4.394277 | 9.634766 | 6.34E-20 | 7.58E-19 | 34.48651 |
| FOXM1 | 1.328435 | 4.718834 | 12.0452 | 8.75E-29 | 6.00E-27 | 54.60333 |
| SERPINB4 | 1.331562 | 2.675074 | 4.838905 | 1.86E-06 | 4.78E-06 | 4.207614 |
| TYMP | 1.337431 | 5.957075 | 8.078019 | 7.57E-15 | 4.64E-14 | 22.99865 |
| SOX9 | 1.344345 | 4.180844 | 7.397109 | 8.04E-13 | 3.97E-12 | 18.42913 |
| S100A9 | 1.349573 | 10.24212 | 4.534082 | 7.62E-06 | 1.83E-05 | 2.858019 |
| ICAM1 | 1.35238 | 4.632162 | 8.363686 | 9.81E-16 | 6.74E-15 | 25.00246 |
| SAA1 | 1.356942 | 3.846598 | 4.862527 | 1.66E-06 | 4.30E-06 | 4.315474 |
| ATP1A1 | 1.35747 | 5.561372 | 11.85965 | 4.56E-28 | 2.61E-26 | 52.97212 |
| WARS1 | 1.360422 | 6.047132 | 9.366779 | 5.21E-19 | 5.40E-18 | 32.41393 |
| PSMB9 | 1.361248 | 5.200023 | 8.234909 | 2.48E-15 | 1.62E-14 | 24.09302 |
| SAMD9L | 1.368284 | 3.233082 | 10.29197 | 3.12E-22 | 5.43E-21 | 39.72043 |
| OAS2 | 1.374914 | 5.212207 | 9.543434 | 1.30E-19 | 1.50E-18 | 33.77601 |
| LYZ | 1.375567 | 5.186147 | 6.469643 | 2.83E-10 | 1.06E-09 | 12.70727 |
| USP18 | 1.379739 | 3.60367 | 13.47882 | 1.73E-34 | 6.68E-32 | 67.57621 |
| EPSTI1 | 1.383467 | 3.92346 | 9.513793 | 1.65E-19 | 1.86E-18 | 33.54633 |
| MX1 | 1.384316 | 5.008029 | 9.946336 | 5.24E-21 | 7.31E-20 | 36.94178 |
| IFI27 | 1.388855 | 6.789765 | 6.47774 | 2.69E-10 | 1.01E-09 | 12.75457 |
| CD274 | 1.390957 | 2.415203 | 9.728558 | 3.01E-20 | 3.79E-19 | 35.22054 |
| IFIT1 | 1.391693 | 3.557976 | 8.076448 | 7.65E-15 | 4.68E-14 | 22.98778 |
| SERPINB3 | 1.394969 | 3.077074 | 4.692301 | 3.70E-06 | 9.22E-06 | 3.548702 |
| LAMC2 | 1.404833 | 5.578414 | 6.616195 | 1.16E-10 | 4.53E-10 | 13.57089 |
| IFI6 | 1.405917 | 8.978259 | 7.364537 | 9.97E-13 | 4.87E-12 | 18.21812 |
| SLC16A1 | 1.416111 | 4.090144 | 7.439092 | 6.08E-13 | 3.04E-12 | 18.70215 |
| LAMA3 | 1.421267 | 3.167507 | 6.947846 | 1.48E-11 | 6.39E-11 | 15.58225 |
| SECTM1 | 1.427617 | 4.082698 | 8.186427 | 3.51E-15 | 2.25E-14 | 23.75323 |
| LAMP3 | 1.430549 | 3.670957 | 11.45293 | 1.64E-26 | 6.47E-25 | 49.43992 |
| IFIH1 | 1.443288 | 3.774888 | 13.21932 | 1.95E-33 | 5.81E-31 | 65.18364 |
| MCM2 | 1.451239 | 4.406559 | 12.65751 | 3.45E-31 | 4.86E-29 | 60.06809 |
| DSC2 | 1.455467 | 3.302645 | 8.481858 | 4.16E-16 | 2.96E-15 | 25.84574 |
| SAMD9 | 1.456213 | 3.739289 | 10.01611 | 2.98E-21 | 4.37E-20 | 37.49814 |
| DSP | 1.466198 | 6.469237 | 7.99043 | 1.40E-14 | 8.34E-14 | 22.3943 |
| OAS3 | 1.469453 | 5.423064 | 13.93774 | 2.29E-36 | 1.85E-33 | 71.84952 |
| GBP5 | 1.487009 | 2.313824 | 8.329043 | 1.26E-15 | 8.51E-15 | 24.75682 |
| IFI44 | 1.491631 | 5.467583 | 9.040625 | 6.43E-18 | 5.75E-17 | 29.94256 |
| RSAD2 | 1.496169 | 3.314659 | 9.674665 | 4.62E-20 | 5.64E-19 | 34.79822 |
| DDX60 | 1.508915 | 3.750355 | 12.03313 | 9.74E-29 | 6.63E-27 | 54.49687 |
| AIM2 | 1.516547 | 3.728944 | 6.74779 | 5.18E-11 | 2.11E-10 | 14.3596 |
| CASP14 | 1.521321 | 3.261274 | 5.014489 | 7.96E-07 | 2.14E-06 | 5.020471 |
| IFI44L | 1.541169 | 3.014148 | 9.821667 | 1.43E-20 | 1.88E-19 | 35.95358 |
| GBP1 | 1.553258 | 5.210692 | 10.54688 | 3.76E-23 | 7.65E-22 | 41.80508 |
| PDZK1IP1 | 1.564396 | 4.252198 | 6.226375 | 1.19E-09 | 4.21E-09 | 11.30857 |
| IFIT3 | 1.584395 | 5.154532 | 9.636576 | 6.25E-20 | 7.49E-19 | 34.50063 |
| IGF2BP2 | 1.589558 | 3.054946 | 8.410152 | 7.01E-16 | 4.89E-15 | 25.33305 |
| DSG3 | 1.611902 | 2.508741 | 5.58481 | 4.29E-08 | 1.31E-07 | 7.835096 |
| S100A7 | 1.6395 | 4.288032 | 4.187232 | 3.46E-05 | 7.69E-05 | 1.419178 |
| STAT1 | 1.64156 | 6.09921 | 14.17888 | 2.31E-37 | 2.46E-34 | 74.11505 |
| IDO1 | 1.655209 | 2.983869 | 7.701077 | 1.04E-13 | 5.61E-13 | 20.43213 |
| KRT6B | 1.685537 | 2.72836 | 4.982529 | 9.30E-07 | 2.47E-06 | 4.870604 |
| KRT5 | 1.686702 | 7.035507 | 4.16778 | 3.76E-05 | 8.31E-05 | 1.341583 |
| CXCL11 | 1.717146 | 3.148402 | 7.794286 | 5.48E-14 | 3.05E-13 | 21.05838 |
| KLHDC7B | 1.752846 | 4.345777 | 6.945478 | 1.50E-11 | 6.48E-11 | 15.56761 |
| S100A8 | 1.781542 | 7.298079 | 5.253468 | 2.41E-07 | 6.82E-07 | 6.167756 |
| TAP1 | 1.782405 | 5.879111 | 13.0124 | 1.32E-32 | 3.13E-30 | 63.28897 |
| CXCL9 | 1.844026 | 4.066863 | 7.710203 | 9.75E-14 | 5.29E-13 | 20.4932 |
| PI3 | 2.050151 | 5.120285 | 5.456776 | 8.44E-08 | 2.50E-07 | 7.180382 |
| CXCL10 | 2.174065 | 5.181924 | 8.997505 | 8.92E-18 | 7.86E-17 | 29.62015 |
| KRT6A | 2.372078 | 5.597845 | 5.603714 | 3.87E-08 | 1.19E-07 | 7.932863 |
